# Human researchers are superior to large language models in writing a medical systematic review in a comparative multitask assessment

**DOI:** 10.1038/s41598-025-28993-5

**Published:** 2025-12-01

**Authors:** Martina Sollini, Cristiano Pini, Alexandra Lazar, Fabrizia Gelardi, Gaia Ninatti, Matteo Bauckneht, Arturo Chiti, Margarita Kirienko

**Affiliations:** 1 Vita-Salute San Raffaele University, Via Olgettina 58, Milan, 20132 Italy; 2 Department of Nuclear Medicine, IRCCS Ospedale San Raffaele, Milan, 20132 Italy; 3 School of Medicine and Surgery, University of Milano-Bicocca, Monza, Italy; 4 Department of Health Science (DISSAL), University of Genoa, Genoa, Italy; 5 Department of Nuclear Medicine, IRCCS Ospedale Policlinico San Martino, Genoa, Italy; 6 Department of Nuclear Medicine, Fondazione IRCCS Istituto Nazionale dei Tumori di Milano, Milan, Italy

**Keywords:** Artificial intelligence, Large language models, Scientific writing, Generative artificial intelligence, Evidence-based medicine, Systematic review, Literature mining, Machine learning, Oncology, Medical research

## Abstract

The capability of Large Language Models (LLMs) to support and facilitate research activities has sparked growing interest in their integration into scientific workflows. This paper aims to evaluate and compare against human researchers the performance of 6 different LLMs in conducting the various tasks necessary to produce a systematic literature review. The evaluation of the 6 LLMs was split into 3 tasks: literature search, article screening and selection (task 1); data extraction and analysis (task 2); final paper drafting (task 3). Their results were compared with a human-produced systematic review on the same topic, serving as reference standard. The evaluation was repeated on two rounds to evaluate between-version changes and improvements of LLMs over time. Out of the 18 scientific articles to be extracted from the literature for task 1, the best LLM managed to identify 13. Data extraction and analysis for task 2 was only partially accurate and cumbersome. The full papers generated by LLMs for task 3 were short and uninspiring, often not fully adhering to the standard PRISMA 2020 template for a systematic review. Currently, LLMs are not capable of conducting a scientific systematic review in the medical domain without prompt-engineering strategies. However, their capabilities are advancing rapidly, and, with an appropriate supervision they can provide valuable support throughout the review process.

**Supplementary Information:**

The online version contains supplementary material available at 10.1038/s41598-025-28993-5.

## Introduction

Large language models (LLMs) are driving a paradigm shift across various disciplines, including health care, education, and research. Ever since the public launch of OpenAI’s ChatGPT in 2022, LLMs have attracted substantial public attention, changing the way we perform certain everyday tasks, as mundane as writing emails. Built on transformer-based deep learning architectures, they are trained on extensive text datasets to learn linguistic patterns, contextual relationships, and semantic meaning, enabling them to generate coherent and relevant writings. Using self-attention mechanisms^1^, LLMs can analyse and predict words based on surrounding context^2^. Furthermore, these models initially pretrained on large datasets, can then be fine-tuned^3^ for specific applications. Their architecture, containing billions of trainable parameters, optimises them for natural language processing, facilitating text interpretation and generation. Notable examples like GPT-4 and BERT have demonstrated significant advancements, leveraging vast datasets and sophisticated algorithms to assist health care professionals^4,5^. GPT-4 has been utilized for tasks like annotation in single-cell RNA sequencing data analysis^6^. In medical imaging, LLMs can automate and enhance various reporting-related tasks, including drafting medical reports and summarising content^5^. Beyond basic applications, LLMs are having an impact on education; for instance, Khan Academy has piloted “Khanmigo” a GPT-4-powered tool designed to guide students through their studies without directly providing answers, thereby promoting active learning^7^.

In research, LLMs have also been employed to assist in various tasks, including literature summarisation and data analysis, thereby accelerating various processes^8^. A recent publication by Gao et al.^9^ even suggests that LLMs are able to write believable scientific abstracts, although with completely generated data, that can trick an unexperienced human reader. While LLMs offer immense potential, it is crucial to address challenges such as ensuring data consistency, mitigating biases, and maintaining transparency in their applications. Responsible integration of these models is essential to fully harness their benefits across health care, education, and research domains.

Despite the growing use of LLMs in research, a direct comparison of their performance against human researchers in conducting a scientific literature review remains underexplored, even though it would hold substantial relevance for the scientific community. Indeed, on one hand a literature review may appear to be the perfect case study of scientific writing for LLMs, since it selects, analyses, and summarises existing large textual datasets of previously published literature. On the other hand, systematic reviews represent the highest level of hierarchy in evidence-based medicine, with the ultimate goal of generating robust evidence on a particular topic. This paper aims to evaluate and compare the capabilities of different LLMs in conducting a systematic literature review. Through a methodical and repeated examination of all the steps needed to conduct a systematic review – from literature search to full paper writing – this study aims to identify the strengths and limitations of different LLMs in automating and enhancing the systematic literature review process.

## Methods

We evaluated and compared the capabilities of six different LLMs in conducting a systematic literature review on alpha-emitting radioligand therapy for metastatic prostate cancer: ChatGPT by OpenAI, Claude by Anthropic, Gemini by Google, DeepSeek R1 by DeepSeek, Le Chat by Mistral, and Grok by xAI. We employed the “plus” plan of ChatGPT and the “pro” plan of Claude; for the other LLMs, the free versions did not present significant limitations to their capabilities, quality, or speed compared to the paid subscription versions for what concerned our evaluations.

We compared the results obtained by each LLM to a paper recently published by our group on the same topic, “Time for Action: Actinium-225 PSMA-Targeted Alpha Therapy for Metastatic Prostate Cancer – A Systematic Review and Meta-Analysis”^10^, that served as a benchmark on human performance, data source and reference standard. Utilising a review conducted by our research group provided the advantage of comprehensive access to raw data, enabling direct comparison between LLMs and human researchers across each task of the scientific process, from literature search to manuscript drafting. This approach ensured transparency and allowed for a nuanced evaluation of AI performance in replicating complex, human-driven research tasks.

The evaluation of the performance of the six LLMs, compared to human experts, was divided in three tasks:

- literature search, article screening and selection (Task 1);
- data extraction and analysis from selected articles (Task 2);
- production of the full text of the systematic review scientific paper (Task 3).

Each task was evaluated independently from the others; specifically, to perform task 2 and task 3 we provided each LLM the same ground-truth data coming from the human-produced scientific paper. Consequently, each LLM started from the same input for these tasks, without influencing each LLM’s performance on previous tasks.

All tasks were evaluated twice, independently, to assess between-version performance changes and potential improvements of LLMs over time. The first evaluation round (Round 1) was conducted on February 23, 2025, employing ChatGPT o3-mini-high, Claude Sonnet 3.7 with Extended Thinking, Google Gemini 2.0 Flash Thinking Experimental with Apps, DeepSeek-R1, and Mistral Le Chat. The second evaluation round (Round 2) was conducted on April 25, 2025, testing ChatGPT o4-mini-high*, Claude Sonnet 3.7 with Extended Thinking, Gemini 2.5 Pro with Deep Research*, DeepSeek-R1, Mistral Le Chat*, and Grok. In the second round, we extended the analyses to Grok, and three LLMs (marked with *), which experienced major updates since the first round of research on February 23, 2025.

### Prompting protocol

Our a priori goal was to evaluate LLMs as they would be used by teams without prompt-engineering expertise. We therefore adopted a minimal, non-optimised prompting protocol designed to replicate the use-case scenario of non-expert teams interacting with LLMs for these research tasks.

For each task, we used a single generic task prompt crafted to be journal-agnostic and novice-friendly (the full texts are available in Supplementary Materials). We avoided exemplar (“few-shot”) templates, chain-of-thought scaffolds, and tool-specific “expert” prompt patterns.

Subsequent turns followed a pre-specified minimal-intervention playbook: (i) restate the original task and the inclusion/exclusion criteria verbatim; (ii) ask the model to persist these criteria for the session; (iii) request corrections of hallucinated citations or formatting errors; (iv) request re-runs when outputs were truncated or incomplete, or in case of server errors. No model-specific hints, exemplars, or optimised rewrites were introduced.

Since prompt wording can have a profound impact on performance, fixing a simple baseline prompt and a uniform adaptation playbook reduces confounding and reflects likely real-world use by teams without specialist prompting skills. This choice trades performance for external validity and transparency. Moreover, it provides the baseline evaluation prior to scaling to an expert-level prompting strategy.

### Task 1

We asked each LLM to retrieve scientific articles related to actinium-PSMA therapy in prostate cancer from the PubMed database, and to screen them applying the same inclusion and exclusion criteria we adopted in the original, human-conducted review^10^. The initial query was the same for each LLM; after that, subsequent interactions followed the above-specified minimal-intervention playbook without introducing model-specific hints or optimised rewrites, to obtain the best possible performance from each LLM without providing “unfair” human assistance. Papers extracted from each LLM were counted and discriminated between real papers meeting inclusion and exclusion criteria, real papers not meeting the criteria, and hallucinated papers.

### Task 2

We uploaded all 18 articles included in the human-conducted review (the ground-truth reference standard^10^ and asked each LLM to extract relevant information from each paper, summarising the findings in a table. LLMs were requested to detect study design, radiopharmaceuticals, baseline patients’ characteristics, follow-up data, and main treatment results. The data tables produced by each LLM was compared against the reference standard, noting for each entry if there was full concordance, partial concordance for partially incorrect or partially missing data, or completely incorrect or missing data.

ChatGPT is the only LLM, at the time of last analysis, that allowed for the construction of a custom GPT partially built on predefined user-submitted documents in a RAG-based approach. We built a custom GPT enriched with scientific documents on how to perform QUADAS and ROBINS quality analysis, available here: https://chatgpt.com/g/g-aVMV7ejsa-systematic-review-assistant. We then performed an additional analysis as a single subtask, evaluating the performance of such a custom GPT in assigning ROBINS-E scores for the 18 selected articles, comparing the results to human assessments using Cohen’s Kappa.

### Task 3

Each LLM was provided with the 18 selected articles, and human-extracted results and analyses, in the form of three tables and seven figures contained in the published reference paper^10^. Each LLM was asked to produce the full scientific paper of a systematic review based on the provided data, adhering to the standard template and constraints of scientific papers and systematic reviews. The content produced by LLMs was qualitatively evaluated by five independent reviewers (CP, MS, MK, MB, AL), and each section (from the title to the references) scored as appropriate, inappropriate, or partially appropriate based on majority voting. The appropriateness of each scientific section was determined by comparing the reported information with the reference standard^10^. A single consistent or inconsistent datum was sufficient to classify a section as partially appropriate. A concordance of 70% or more with the reference standard was considered appropriate, while if more than 50% of a section’s content differed from expectations it was deemed inappropriate. The length of each section was also recorded.

We referred to the “standard template” for a systematic review as the PRISMA 2020 reporting guideline (27-item checklist) and the PRISMA 2020 extension for Abstracts^11^. We used these checklists to define the sections and minimum information expected in a complete systematic review draft. Items mapped as follows: Title (PRISMA item 1; Abstract items 1–12), Introduction (items 3–4), Methods (items 5–15: protocol/registration, eligibility criteria, information sources, full search strategy, selection and data-collection processes, data items, risk-of-bias assessment, effect measures, synthesis methods, reporting-bias and certainty assessments), Results (items 16–22: study selection with PRISMA flow, study characteristics, risk of bias in studies, results of individual studies and syntheses), Discussion (item 23: interpretation and limitations at evidence and review level), and References.

### Qualitative evaluation and time effectiveness

For all tasks, we assigned each LLM a qualitative grading scale on their performance (green: good; yellow: average; red: unsatisfactory; grey: not assessable), considering the second round of evaluation. All gradings were collectively assigned by all authors, and developed on the basis of the quantitative evaluations for each Taks detailed in the previous paragraphs, integrated by qualitative subjective judgements on LLMs output.

We also tracked the time needed to complete each task by the LLMs, and compared these data to the human-hours needed to complete them for the production of the reference-standard systematic review.

## Results

Queries and LLMs outputs are provided as Supplementary Material.

### Task 1

In the original systematic review^10^, 4,362 references were initially identified, 24 records were assessed for eligibility, and, ultimately, 18 studies that met the specified criteria were selected and analysed.

Table 1 summarises the results of Task 1 related to article search, screening, and selection.

In Round 1, ChatGPT (o3-mini-high) outperformed the other LLMs, correctly identifying 8 papers, alongside one article not meeting selection criteria and several hallucinated papers. Notably, multiple subsequent attempts were needed to generate a list containing more than 3–4 papers; it is although worth noting that the shorter lists generated at first did not contain hallucinated articles. Gemini (2.0 Flash Thinking Experimental with Search) at first produced only a few hallucinated papers and, similarly to ChatGPT, several attempts were made to obtain longer lists of papers which only comprised hallucinated articles, reviews, or manuscripts not meeting criteria. Overall, in Round 1 all LLMs also identified papers not meeting the selection criteria, including reviews, editorials, pre-clinical trials, duplicates, study design presentation, and off-topic papers.

In Round 2, the updated version of ChatGPT (o4-mini-high) improved, correctly selecting 9 papers, without additional incorrect articles. Gemini (2.5 Pro Experimental) demonstrated a relevant improvement over its previous version and outperformed the other LLMs, identifying 13 correct papers meeting the inclusion and exclusion criteria, and no other articles. Gemini also autonomously provided, unprompted, a data table summarising findings from the identified papers and conceived a short paper, partially fulfilling Task 2 and Task 3. Both LLMs managed to produce these lists at their first attempts, without improvements after additional prompts. Grok managed to select 8 correct papers, while also providing one hallucinated manuscript.

In both rounds, Claude (3.7 Sonnet with Extended Thinking) explicitly stated that it cannot complete the task as it does not have access to PubMed or other similar web databases. DeepSeek and Mistral generally underperformed compared to Gemini, ChatGPT and Grok, with DeepSeek experiencing at times severe server errors that hindered the analysis.

**Table 1:** Task 1 results.

	Correct Papers, (*n*)	Papers not meeting inclusion/exclusion criteria (*n*)	Hallucinated papers (*n*)
**Round 1 – Feb 2025**			
ChatGPT o3-mini-high	8	1	10
Claude Sonnet 3.7 with Extended Thinking	n.a.	n.a.	n.a.
Google Gemini 2.0 Flash Thinking Experimental	0	4	30
DeepSeek R1	5	25	3
Mistral Le Chat	2	7	0
**Round 2 – Apr 2025**			
ChatGPT o4-mini-high	9	0	0
Claude Sonnet 3.7 with Extended Thinking	n.a.	n.a.	n.a.
Google Gemini 2.5 Pro Experimental	13	0	0
DeepSeek R1	2	0	0
Mistral Le Chat	3	1	0
Grok 3	8	0	1

Summary of the results of Task 1 – article search, screening and selection – in the two rounds of evaluation.

### Task 2

The reference standard was represented by the Table “Summary of baseline characteristics and outcomes of studies included in the systematic review and meta-analysis” in the human-conducted article^10^, that included 11 entries for each paper for a total of 198 entries overall, that were compared with the tabulated results for each LLM.

Table 2 summarizes the results of Task 2.

In Round 1, Claude outperformed other LLMs, correctly compiling 92% of table entries, with 9 out of 18 articles presenting all data correctly tabulated. Notably, the task was particularly cumbersome and time consuming, as manuscripts could only be uploaded and analysed 3 or 4 at a time. Mistral Le Chat also managed to obtain similar, even though more partial results (88.9% of entries, 6/18 completely correct articles) – with analogous limitations on the upload and analysis procedure. Gemini and DeepSeek were not capable of adequately performing Task 2, due to functional limitations or to multiple server-side errors and halts, respectively.

In Round 2, Claude and Mistral demonstrated a slight underperformance compared to Round 1, still maintaining a correct entry rate of respectively 81.8% and 79.3%. Gemini managed 91% correct entries, reaching 7 out of 18 completely correct papers. DeepSeek was the best performer of Round 2 in terms of correct entries (93%), with 7 out of 18 fully correct papers, although this LLM still experienced some minor server issues and it required a complex procedure of upload and analysis of one or two articles at a time. Grok could not be adequately tested for Task 2, as the built-in web search capabilities lead the LLM to retrace from the internet our very ground-truth article and to present the related reference-standard Table.

The subtask of assessing the ROBINS-E risk of bias, performed only by our home-made custom GPT in ChatGPT resulted in a poor agreement between the LLM and the reference-standard^10^ (0.556), with a Cohen’s Kappa of 0.286. The full data table for this subtask in available in Supplementary Materials.

**Table 2:** Task 2 results.

	Data entries, *n* (%)			Completely correct articles
	Correct	Partially Correct or Partially Missing Data	Wrong	
**Round 1 – Feb 2025**				
ChatGPT o3-mini-high	86 (43.4%)	13 (6.6%)	99 (50%)	0/18
Claude Sonnet 3.7 with Extended Thinking	183 (92.4%)	11 (5.6%)	4 (2%)	9/18
Google Gemini 2.0 Flash Thinking Experimental	n.a.	n.a.	n.a.	n.a.
DeepSeek R1	n.a.	n.a.	n.a.	n.a.
Mistral Le Chat	176 (88.9%)	17 (8.6%)	5 (2.5%)	6/18
**Round 2 – Apr 2025**				
ChatGPT o4-mini-high	99 (50%)	17 (8.6%)	82 (41.4%)	0/18
Claude Sonnet 3.7 with Extended Thinking	162 (81.8%)	22 (11.1%)	14 (7.1%)	3
Google Gemini 2.5 Pro Experimental	180 (90.9%)	15 (7.6%)	3 (1.5%)	7
DeepSeek R1	185 (93.4%)	13 (6.6%)	0 (0%)	7
Mistral Le Chat	157 (79.3%)	23 (11.6%)	8 (4%)	3
Grok 3	n.a.	n.a.	n.a.	n.a.

Summary of the results of Task 2 – selected article analysis and data extraction – in the two rounds of evaluation.

### Task 3

The ground truth article^10^ comprised seven sections (title, abstract, introduction, methods, results, discussion including conclusions, and references). The four sections reporting information as required by the specific journal’s editorial policy (acknowledgements, funding, data availability, and competing interests) were excluded from this analysis. The total number of references included in the paper was 46, while the word count for the title, abstract, and main text was 16, 268, and 4252, respectively (466 for the introduction, 880 for the methods, 1535 for the results, and 1371 for the discussion, respectively)^10^. A summary of the evaluation of the drafts produced by each LLMs is presented in Table 3. Supplementary Table 1 further details the content of each paper according alongside synthetic comments from the evaluation.

In both Rounds, no LLM reached satisfactory results. Claude was the best performing LLM, and notably the only one producing a reference section. ChatGPT in Round 1 did provide a disclaimer stating: “References to the individual studies included in the analysis are available upon request and should be formatted according to the target journal’s guidelines”. All the other manuscript sections were included by all LLMs.

Regarding writing accuracy, the title was the only section deemed as completely appropriate in 2/5 cases in Round 1 and in 5/6 LLMs in Round 2, while all other sections were classified as either partially appropriate or inappropriate. The length of all LLM-generated drafts was greatly shorter than the reference standard^10^, with a total word count of the main text ranging from 487 for DeepSeek to 4252 for Claude.

**Table 3:** Task 3 results.

	Title	Abstract	Introduction	Methods	Results	Discussion	References
**Round 1 – Feb 2025**							
ChatGPT o3-mini-high	**A**	**P.A.**	**P.A.**	**I**	**I**	**I**	**I**
Claude Sonnet 3.7 with Extended Thinking	**P.A.**	**P.A.**	**P.A.**	**P.A.**	**P.A.**	**P.A.**	**P.A.**
Google Gemini 2.0 Flash Thinking Experimental	**P.A.**	**P.A.**	**P.A.**	**I**	**P.A.**	**P.A.**	**I**
DeepSeek R1	**P.A.**	**P.A.**	**I**	**I**	**I**	**I**	**I**
Mistral Le Chat	**A**	**P.A.**	**I**	**I**	**I**	**I**	**I**
**Round 2 – Apr 2025**							
ChatGPT o4-mini-high	**I**	**P.A.**	**I**	**I**	**I**	**I**	**I**
Claude Sonnet 3.7 with Extended Thinking	**P.A.**	**P.A.**	**P.A.**	**P.A.**	**P.A.**	**P.A.**	**P.A.**
Google Gemini 2.5 Pro Experimental	**P.A.**	**P.A.**	**P.A.**	**I**	**P.A.**	**P.A.**	**I**
DeepSeek R1	**P.A.**	**P.A.**	**I**	**I**	**I**	**I**	**I**
Mistral Le Chat	**A**	**P.A.**	**I**	**I**	**I**	**I**	**I**
Grok 3	**A**	**P.A.**	**P.A.**	**I**	**I**	**I**	**I**

Summary of the results of Task 3 – final manuscript production – in the two rounds of evaluation. A: appropriate; P.A.: partially appropriate; I: inappropriate.

### Qualitative evaluation and time effectiveness

Table 4 reports an overall quality judgement on LLMs performance on each task, alongside the approximate time needed to complete them.

For the human-conducted systematic review, researchers took approximately 50, 4, and 30 h to complete Task 1, Task 2, and Task 3, respectively.

Using LLMs, Task 1 and Task 3 where generally accomplished quickly with all models managing to complete them in 30 min or less. Task 2 required cumbersome time-consuming procedures for many of the models.

**Table 4:** Overall evaluation on quality and time-effectiveness.

	Task 1	Task 2	Task 3
ChatGPT o4-mini-high	$\color{yellow} {\bullet}$/20 min	$\color{red} {\bullet}$/15 min	$\color{red} {\bullet}$/5 min
Claude Sonnet 3.7 with Extended Thinking	${\bullet}$/n.a.	$\color{green} {\bullet}$/90 min	$\color{yellow} {\bullet}$/5 min
Google Gemini 2.5 Pro Experimental	$\color{green} {\bullet}$/5 min	$\color{green} {\bullet}$/15 min	$\color{yellow} {\bullet}$/5 min
DeepSeek R1	$\color{red} {\bullet}$/30 min	$\color{green} {\bullet}$/120 min	$\color{red} {\bullet}$/5 min
Mistral Le Chat	$\color{red} {\bullet}$/5 min	$\color{yellow} {\bullet}$/90 min	$\color{red} {\bullet}$/5 min
Grok 3	$\color{yellow} {\bullet}$/5 min	${\bullet}$/n.a.	$\color{red} {\bullet}$/5 min

Overall evaluation of the quality of results on each Task (green: good; yellow: average; red: bad), with approximate time needed to execute each Task.

## Discussion

This comparative study between 6 LLMs and human experts revealed limitations in the current capacity of LLMs to independently perform a scientific systematic literature review. Applying a task-based step-by-step approach, we proved that LLMs underperformed in key tasks, such as literature search, data summary, and manuscript drafting. In summary, Gemini demonstrated the best performance in Task 1 with quick retrieval of 13/18 correct articles alongside one hallucinated paper. DeepSeek resulted to perform best at Task 2 with overall 93% correct entries, and with fully correct paper entries in 7 out of 18 cases. In manuscript drafting no LLM reached satisfactory results.

Specifically, in Task 1, five out of six LLMs engaged in literature search and selection. Even applying iterative prompting, LLMs were not able to yield a comparable number of relevant articles to the established reference standard in either Round 1 or Round 2 (Table 1). This can be related to the fact that LLMs don’t inherently use or reveal structured querying and search strategy. Moreover, some LLMs might not have direct, real-time access to academic databases (e.g., PubMed, Embase, Scopus) unless explicitly integrated with external tools or plugins. Finally, scientific articles may be underrepresented among training data for LLMs compared to other textual productions, so LLMs may apply too general heuristics missing context-specific cues. Nevertheless, while results for Task 1 were not satisfactory enough, LLMs did manage to extract several appropriate papers in a much shorter amount of time compared to human researchers. This capability can be exploited for a first scan of the scientific literature. It can also be postulated that such a “LLM-assisted search” can be already integrated into the workflow of paper retrieval for a systematic review, alongside the standard human-conducted cross-search of databases and references.

In contrast to previous studies^12,13^, our analysis revealed a relative underperformance of LLMs in Task 2, which consisted of extracting data from the selected papers in a structured manner. The output generated by LLMs frequently resulted in missing data or wrong information across many input articles. At most 9 out of 18 articles were tabulated with fully correct entries, not requiring additional human intervention. Furthermore, three good-performing LLMs in Task 2 required slow and complex prompting strategies and multiple uploads to obtain the results, demonstrating low time-efficiency compared to human work. The difference between our findings and published data might be related to the use of different models, versions, implementations, and interactions. Indeed, in a recent study Li et al.^14^ demonstrated the effectiveness of guided prompting strategies (such as knowledge-guided prompting), in enhancing the performance of LLMs in several review tasks, e.g. PICO items extraction (problem, intervention, comparison, outcome). Beyond those findings, several studies show that prompt formulation and formatting can substantially alter downstream outcomes^15–17^ supporting the need to report and justify prompt choices. Our study therefore reports initial prompts verbatim and details a standardised subsequent adaptation playbook, but by design did not pursue optimised prompts. The present study specifically aimed to test the inherent capabilities of LLMs in conducting a systematic review through simple and unstructured interactions, easily reproducible by researchers with no specific background on LLMs or LLM prompting.

In the more structured subtask related to the risk of bias assessment performed by our ChatGPT-based custom GPT, we found poor agreement between the LLM and humans (Cohen’s Kappa of 0.286). This discrepancy might be related to the restrained ability of the model to interpret nuanced methodological details and contextual cues from scientific literature: LLMs can summarise and interpret text but lack full contextual judgment.

Unexpectedly, LLMs performed poorly in Task 3, even though this task is apparently more adherent to their text-generating capabilities. In Round 1, LLMs produced wrong titles, for example misclassifying the specific clinical setting of the systematic review. In Round 2, the generated titles were acceptable, albeit strikingly similar among LLMs and uninspiring. All the remaining sections were generally extremely short with inappropriate or partially appropriate content. Consistently with this, the drafts frequently missed core PRISMA 2020 elements, rendering the outputs structurally incomplete even when the prose appeared polished. These findings are in line with previous investigations reporting similar limitations^18^. Regarding the formal aspects, LLMs provided text with well-structured format and correct scientific language, which can be misleading for non-expert readers. In this scenario, critical evaluation of published literature by human experts remains essential to assess methodological limitations, potential biases, and the strength of evidence. Indeed, systematic reviews and meta-analyses are considered the gold standard in evidence-based medicine^19^, and the selection and correct evaluation of high-quality source publications is essential to guide clinical practice.

There are no widely accepted benchmarks or metrics for comparative analyses between LLMs and human-authored content. There are some task-specific benchmarks or evaluation frameworks, such as BLEU^20^, ROUGE^21^, METEOR^22^, for evaluating text similarity, mostly in machine translation and summarization. TruthfulQA^23^, BIG-Bench^24^, and MMLU^25^ are benchmarks for testing factual accuracy, reasoning, or domain knowledge in LLMs. Turing test-style human evaluations compare artificial intelligence versus human output in subjective quality, coherence, or relevance assessment^26^. However, these are limited in scope, often focusing on short-form tasks, general knowledge, or artificial scenarios. On the other hand, human content itself is variable in quality. There is no universal benchmark defining what makes human writing “correct” or “better.” In the present study, we applied qualitative multi-reader scores, considering that human experts are able to provide traceable justifications for their decisions.

The scientific community has developed some initiatives^27–29^, including the EQUATOR Network^30^, to guide authors in using explicit methodologies and reporting research. Adhering to EQUATOR reporting guidelines during research development and article writing enhances the reliability and value of published health research literature by promoting transparent and accurate reporting^30^. Notably, the systematic review we used as reference^10^ followed the PRISMA reporting guidelines^11^, a framework that also ensures results can be replicated by the scientific community. Systematic reviews require documentation for reproducibility (e.g., PRISMA diagrams, search logs). LLMs do not track, log, or document their “search path” in a structured way unless explicitly prompted to simulate it^15^.

The repetition of our full analyses on all tasks in two rounds, two months apart, highlighted the inherent inconsistency of all LLMs evaluations and revealed marked between-version variability. It is worth recognising that, under widely used definitions, “reproducibility” requires holding data, code, methods, and computational conditions constant^31^ our two-round evaluation therefore does not represent a reproducibility study but a version-update comparison intended to characterise temporal changes. Nonetheless, general trends are worth noting: between Round 1 and Round 2, all LLMs mostly improved – in some cases by a wide margin. Remarkably, a significant reduction of confabulations and hallucinations, which are well-known limitations affecting LLMs since their inception, was observed.

The accomplishment of several tasks resulted time-consuming and cumbersome, with potential influence on energy and natural resources consumption, as LLMs require significant computational resources, energy, and materials. On the other hand, scientific research conducted by humans is not without environmental cost, as it involves prolonged use of computational devices, internet, and human resources that also contribute to CO₂ emissions. In this regard, some data comparing the power consumption of machine learning algorithms versus human activity suggest that AI, when faster – a condition not always met in our Tasks –, might be environmentally beneficial^32,33^. The opposing narratives, framing LLMs as either a threat or a remedy to sustainability, highlight the urgent necessity of rigorously assessing their environmental footprint compared to that of human activity. Future advancements, such as the move towards quantum computing, may significantly shift the balance towards a less impactful footprint of generative artificial intelligence^34^.

We acknowledge there are some limitations to our investigation. Firstly, we used one single systematic review for comparison. This was intentionally selected because we had access to all the raw data related to the paper, as the manuscript has undergone the peer review process and has been published open-access, ensuring transparency and granting the validity of the publication. This single-paper, single-domain design limits generalisability and may embed domain-specific patterns that do not extrapolate directly to other fields. It also raises the possibility of self-assessment bias when comparing LLM outputs to a publication authored by members of the present team. However, LLM were also evaluated by three reviewers who were not authors of the reference systematic review, and we relied on predefined, task-specific quantitative criteria. Nevertheless, residual bias cannot be excluded. Future studies should evaluate multiple systematic reviews across diverse biomedical (and non-biomedical) domains, authored by independent teams, with pre-registered protocols and cross-site assessors, to improve robustness and external validity^35,36^. Finally, we did not systematically vary or optimise prompts nor compare alternative phrasings, templates, or scaffolded strategies.

Performance under engineered prompts may differ. Our prompting choice was intentional: we prioritised asking what benefit current LLMs offer to a typical research team without prompt-engineering expertise. Accordingly, we fixed simple baseline prompts and applied a uniform minimal-intervention playbook rather than model-specific “expert” prompts or step-by-step scaffolds. This design reduces prompt-induced confounding and aims at making our workflow more representative, at the cost of potential better performance obtainable with engineered prompts. Most research teams lack expertise in prompt engineering. By using simple prompts, our study reflects the actual conditions under which LLMs are likely to be used in academic environments today. Establishing a clear, uniform baseline is essential to evaluate the intrinsic capability of LLMs, independent of human prompt-engineering skill. Research teams often face constraints of time, resources, and technical skill. A simple prompting strategy demonstrates whether LLMs can provide practical utility with low entry barriers. Using simple baselines provides a foundation against which more advanced prompting strategies can later be tested.

This was an explicit decision to reflect “novice”, real-world use and to minimise prompt-induced confounding. However, it likely underestimates the performance achievable with optimised or guided prompting. Future work could incorporate pre-registered prompting protocols, multiverse prompt analyses, and sensitivity analyses that quantify how conclusions change under reasonable prompt variants.

## Conclusion

While LLMs have shown remarkable capabilities in natural language processing and generation, they remain unreliable and markedly inferior to human experts in producing scientific systematic reviews when employed without prompt-engineering strategies. Tasks such as assessing risk of bias, synthesising heterogeneous evidence, and drawing clinically relevant conclusions extend beyond linguistic proficiency. These activities require domain-specific expertise, critical interpretative judgment, and a thorough understanding of research methodology. Nonetheless, when appropriately supervised, LLMs can serve as valuable tools to support researchers in select aspects of the review process.

We are still far from Arthur C. Clarke’s vision: our modern-day HAL 9000 s are still not able to take control over the spaceship. Opponents of LLMs may rejoice, cheering the emerging uncontested winner of our scientific tournament: human researchers. Artificial Intelligence is not capable of independently producing a scientific systematic review – yet.

## Supplementary Information

Below is the link to the electronic supplementary material.

Supplementary Material 1

Supplementary Material 2

Supplementary Material 3

## Data Availability

All relevant data and materials are included in the manuscript and in the supplementary files.
