# Supplementary material for: Human researchers are superior to large language models in writing a medical systematic review in a comparative multitask assessment
Source: Sci Rep. 2025 Dec 1;16:173. doi: 10.1038/s41598-025-28993-5 (PMC12765003; doi:10.1038/s41598-025-28993-5)
Supplement: Supplementary file 1 — Supplementary Material 1 [file 41598_2025_28993_MOESM1_ESM.zip › Supplementary Materials/Round 1/Task 2/Claude 3.7 Data Extraction Table.docx]

Claude 3.7 Sonnet Extended Thinking Mode.

Correct Entries: 183/198 (completely correct articles: 9/18)

Partially Correct Entries or Partially Missing Data: 11/198

Wrong Entries: 4/198

Note: papers to be analysed 4 at a time – not the fastest, not possible to obtain a complete table with all papers

| Ref | Study design | Patients (n) | Age (mean /median) | ECOG PS  (median) | Baseline PSA (median, ng/mL) | Metastases | Prior systemic treatments | Radiopharmaceutical  and treatment regimen | Number of cycles (median) | Follow-up time  (median) | Main results |
| --- | --- | --- | --- | --- | --- | --- | --- | --- | --- | --- | --- |
| Zacherl et al (2020) | Retrospective | 14 | 75 (median) | 1 | 112 | Skeletal: 93%  Lymph node: 71%  Visceral: 21% | ADT: 100%  ARPi: 100%  Taxane-based CT: 86%  [177Lu]Lu-PSMA-617: 79%  Radium-223 dichloride: 14% | [225Ac]Ac-PSMA-I&T  100 kBq/kg every 8 weeks | 2 | 5.4 months | PSA50: 50%  Any PSA reduction: 79%  mPFS: NA  mOS: NA |
| Sathekge et al (2023) | Retrospective | 21 | 67 (median) | 1 | 197 | Skeletal: 100%  Lymph node: NA  Visceral: 29% | None | [225Ac]Ac-PSMA-617  8 MBq followed by de-escalation every 8 weeks | 3 | NA | PSA50: 86%  Any PSA reduction: 95%  mPFS: NA  mOS: 31 months (CI 13-49) |
| Sanli et al (2021) | Retrospective | 12 | 70 (median) | 2 | 129 | Skeletal: 100%  Lymph node: 75%  Visceral: 17% | ADT: 100%  ARPi: 92%  Taxane-based CT: 83%  [177Lu]Lu-PSMA-617: 58%  Radium-223 dichloride: NA | [225Ac]Ac-PSMA-617  100 kBq/kg every 8 weeks | 2 | 10 months | PSA50: 50%  Any PSA reduction: 75%  mPFS: 4 months (CI NA)  mOS: 10 months (CI NA) |
| Sathekge et al (2024) | Retrospective | 488 | 68 (mean) | 1 | 170 | Skeletal: 89%  Lymph node: 72%  Visceral: 20% | ADT: 86%  ARPi: 50%  Taxane-based CT: 67%  [177Lu]Lu-PSMA-617: 32%  Radium-223 dichloride: 4% | Radiopharmaceutical NA  8 MBq followed by de-escalation every 8 weeks | 2 | 9 months | PSA50: 57%  Any PSA reduction: 73%  mPFS: 8 months (CI 7-9)  mOS: 15.5 months (CI 13-18)  (MISSING DATA) |
| Selcuk et al (2023) | Retrospective | 23 | 70 (mean) | NA | 104 | Skeletal: 91%  Lymph node: 56%  Visceral: NA | ADT: 100%  ARPi: 83%  Taxane-based CT: 96%  [177Lu]Lu-PSMA-617: 100%  Radium-223 dichloride: NA | [225Ac]Ac-PSMA-617  100 kBq/kg with a median interval of 13 weeks | 2 | NA | PSA50 (after the 1st cycle): 26%*  Any PSA reduction (after the 1st cycle): 58%*  mPFS: 3 months (CI NA)  mOS: 8 months (CI NA) |
| Lawal et al (2022) | Retrospective | 106 | 69 (mean) | NA | 250 | Skeletal: 100%  Lymph node: 60%  Visceral: 15% | ADT: 100%  ARPi: 13%  Taxane-based CT: 45%  [177Lu]Lu-PSMA-617: 7%  Radium-223 dichloride: 2% | [225Ac]Ac-PSMA-617  8 MBq followed by de-escalation every 8 weeks | 4 | 8 months | PSA50: 80%  Any PSA reduction: NA  mPFS: 14 months (CI 8-20)  mOS: 15 months (CI 13-17) |
| Sathekge et al (2022) | Retrospective | 53 | 63 (median) | 1 | 466 | Skeletal: 89%  Lymph node: 68%  Visceral: 11% | ADT: 100%  ARPi: 0%  Taxane-based CT: 0%  [177Lu]Lu-PSMA-617: 0%  Radium-223 dichloride: 0% | [225Ac]Ac-PSMA-617  8 MBq followed by de-escalation every 8 weeks | 3 | NA | PSA50: 91%  Any PSA reduction: 96%  mPFS: NA  mOS: NA |
| Yadav et al (2020) | Prospective | 28 | 70 (mean) | 3 | 222 | Skeletal: 96%  Lymph node: 86%  Visceral: 32% | ADT: 100%  ARPi: 100%  Taxane-based CT: 93%  [177Lu]Lu-PSMA-617: 54%  Radium-223 dichloride: NA | [225Ac]Ac-PSMA-617  100 kBq/kg every 8 weeks | 3 | 10 months | PSA50: 39%  Any PSA reduction: 89%  mPFS: 12 months (CI 9-13)  mOS: 17 months (CI 16-NR) |
| Ballal et al (2023) | Retrospective | 56 | 68 (median) | 3 | NA | Skeletal: 95%  Lymph node: 95%  Visceral: 43% | ADT: 100%  ARPi: 98%  Taxane-based CT: 89%  [177Lu]Lu-PSMA-617: 48%  Radium-223 dichloride: NA | [225Ac]Ac-PSMA-617  100-150 kBq/kg every 8 weeks | 4 | 22 months | PSA50: 68%  Any PSA reduction: 91%  mPFS: 9 months (CI 7-15)  mOS: 15 months (CI 10-19) |
| Doelen et al (2020) | Retrospective | 13 | 71 (median) | NA | 878 | Skeletal: 100%  Lymph node: 85%  Visceral: 62% | ADT: 100%  ARPi: 85%  Taxane-based CT: 100%  [177Lu]Lu-PSMA-617: 15%  Radium-223 dichloride: 31% | [225Ac]Ac-PSMA-617  8 MBq followed by de-escalation every 8 weeks | 3 | NA | PSA50: 69%  Any PSA reduction: 85%  mPFS: 5.5 months (CI NA)  mOS: 8.5 months (CI NA) |
| Kratochwil et al (2018) | Retrospective | 40 | 70 (median) | 1 | 169 | Skeletal: 98%  Lymph node: NA  Visceral: 40% | ADT: 100%  ARPi: NA%  Taxane-based CT: NA%  [177Lu]Lu-PSMA-617: NA  Radium-223 dichloride: 23% | [225Ac]Ac-PSMA-617  100 kBq/kg every 8 weeks | 3 | NA | PSA50: 73%  Any PSA reduction: 93%  mPFS: 7 months (CI NA)  mOS: NA |
| Sen et al (2021) | Retrospective | 38 | 68 (median) | NA | 147 | Skeletal: 100%  Lymph node: 53%  Visceral: 18% | ADT: 100%  ARPi: 84%  Taxane-based CT: 100%  [177Lu]Lu-PSMA-617: 24%  Radium-223 dichloride: 5% | [225Ac]Ac-PSMA-617  100 kBq/kg every 8 weeks | 2 | 14 months | PSA50: 66%  Any PSA reduction: 87%  mPFS: 8 months (CI 5-10.5)  mOS: 12 months (CI 9-15) |
| Sathekge et al (2018) | Retrospective | 17 | 65 (mean) | 0 | 49 | Skeletal: 82%  Lymph node: 53%  Visceral: 12% | ADT: 65%  ARPi: 0%  Taxane-based CT: 0%  [177Lu]Lu-PSMA-617: 18%  Radium-223 dichloride: 0% | [225Ac]Ac-PSMA-617  8 MBq followed by de-escalation every 8 weeks | 3 | 13 months | PSA50: 88%  Any PSA reduction: 94%  mPFS: NA  mOS: NA |
| Sathekge et al (2019) | Retrospective | 73 | 69 (median) | 0 | 57 | Skeletal: 90%  Lymph node: 58%  Visceral: 8% | ADT: 100%  ARPi: 1%  Taxane-based CT: 37%  [177Lu]Lu-PSMA-617: 14%  Radium-223 dichloride: 1% | [225Ac]Ac-PSMA-617  8 MBq followed by de-escalation every 8 weeks | 3 | 9 months | PSA50: 74%  Any PSA reduction: 82%  mPFS: 15 months (CI 13-17.5)  mOS: 18 months (CI 16-20) |
| Feuerecker et al (2020) | Retrospective | 26 | 73 (median) | 1 | 331 | Skeletal: 100%  Lymph node: 88%  Visceral: 42% | ADT: 100%  ARPi: 100%  Taxane-based CT: 100%  [177Lu]Lu-PSMA-617: 100%  Radium-223 dichloride: 23% | [225Ac]Ac-PSMA-617  Activity and interval NA | 2 | 7 months | PSA50: 65%  Any PSA reduction: 88%  mPFS: 3.5 months (CI 2-11)  mOS: 8 months (CI 4.5-12) |
| Tagawa et al (2023) | Phase I open-label dose escalation trial | 32 | 70 (median) | 1 | 149 | Skeletal: 97%  Lymph node: 88%  Visceral: NA | ADT: 100%  ARPi: 100%  Taxane-based CT: 63%  [177Lu]Lu-PSMA-617: 47%  Radium-223 dichloride: 28% | 225Ac-J591  Single dose, with activity range 13.3-93.3 kBq/kg | 1 | NA | PSA50: 47%  Any PSA reduction: 72%  mPFS: 5.5 months (CI 4-8)  mOS: 11 months (CI 6.5-17) |
| Satapathy et al (2020) | Retrospective | 11 | 68 (median) | 1 | 158 | Skeletal: 100%  Lymph node: 82%  Visceral: 0% | ADT: 100%  ARPi: NA  Taxane-based CT: NA  [177Lu]Lu-PSMA-617: 46%  Radium-223 dichloride: 0% | [225Ac]Ac-PSMA-617  100 kBq/kg every 8-12 weeks | 2 | NA | PSA50: 45%  Any PSA reduction: 73%  mPFS: NA  mOS: NA |
| Rathke et al (2024) | Retrospective | 104 | 62 (median) | 1 | 312 | Skeletal: 96%  Lymph node: 70%  Visceral: NA | ADT: 100%  ARPi: 89%  Taxane-based CT: 70%  [177Lu]Lu-PSMA-617: 37%  Radium-223 dichloride: NA | [225Ac]Ac-PSMA-617  6-8 MBq followed by de-escalation every 8 weeks | 2 | NA | PSA50: 53%  Any PSA reduction: NA  mPFS: NA  mOS: 9 months (CI 7-11) |
