# Supplementary material for: Human researchers are superior to large language models in writing a medical systematic review in a comparative multitask assessment
Source: Sci Rep. 2025 Dec 1;16:173. doi: 10.1038/s41598-025-28993-5 (PMC12765003; doi:10.1038/s41598-025-28993-5)
Supplement: Supplementary file 1 — Supplementary Material 1 [file 41598_2025_28993_MOESM1_ESM.zip › Supplementary Materials/Round 1/Task 1/Database Search v2.docx]

**Risultati ricerca 23/02/2025**

**ChatGPT (o3-mini-high)**

8 correct papers

1 papers not meeting the inclusion criteria

10 hallucinated papers

(note: several attempts need to obtain a list of more than 3-4 papers; these short lists did include however all correct papers).

**Gemini (2.0 Flash Thinking Experimental with Search)**

30 hallucinated papers

(note: several attempts needed to obtain a list of more than 3-4 papers; these short list did also include reviews or papers not meeting inclusion criteria)

**Claude (3.7 Sonnet with Extended Thinking)**

Explicitely states it can not complete the task as it does not have access to PubMed or other similar web databases.

**DeepSeek (R1+search)**

5 correct papers

25 papers not meeting inclusion criteria (9 reviews including our preprint on Theranostics, 1 study design, 1 preclinical paper, 5 off-topic paper, 1 divulgative article, 1 editorial, 7 duplicated correct results)

3 hallucinated papers

**Mistral Le Chat**

2 correct papers

7 papers not meeting inclusion criteria (4 reviews, 2 preclinical papers, 1 editorial)

**Update 20/03/2025 (* = major update since 23/02/2025)**

**ChatGPT (4.5 with Deep Research) ***

11 correct papers

1 paper not meeting inclusion criteria

**Gemini (Deep Research) ***

4 correct papers

1 paper not meeting inclusion criteria (study design)

Gemini provided without request tables with data extracted from the 5 studies identified by the LLM, and a short paper, partially and autonomously performing Task 2 and Task 3; these tables for Task 2, even though only on 5 articles 1 of which to be excluded, were correct in their content.

Unfortunately, even after insisting, Gemini did not select more than these 5 papers.

**Claude (3.7 Sonnet with Extended Thinking)**

Explicitely states it can not complete the task as it does not have access to PubMed or other similar web databases.

**DeepSeek (R1+search)**

(Was not able to perform the search due to “service unavailable” – such inconveniences happen relatively often with this LLM).

**Mistral Le Chat ***

3 correct papers; won’t provide any additional paper even after reiterated requests.
