# Supplementary material for: Human researchers are superior to large language models in writing a medical systematic review in a comparative multitask assessment
Source: Sci Rep. 2025 Dec 1;16:173. doi: 10.1038/s41598-025-28993-5 (PMC12765003; doi:10.1038/s41598-025-28993-5)
Supplement: Supplementary file 1 — Supplementary Material 1 [file 41598_2025_28993_MOESM1_ESM.zip › Supplementary Materials/Round 1/Task 3/Full Paper Claude.docx]

**Efficacy and Safety of Targeted Alpha Therapy with Actinium-PSMA in Metastatic Castration-Resistant Prostate Cancer: A Systematic Review and Meta-Analysis**

**Abstract**

**Background**: Metastatic castration-resistant prostate cancer (mCRPC) represents a significant clinical challenge with limited therapeutic options. Targeted alpha therapy (TAT) with actinium-225-labeled prostate-specific membrane antigen (225Ac-PSMA) has emerged as a promising approach. We performed a systematic review and meta-analysis to evaluate the efficacy and safety of 225Ac-PSMA TAT in patients with mCRPC.

**Methods**: A comprehensive literature search was conducted to identify studies reporting outcomes of 225Ac-PSMA TAT in mCRPC patients. The primary outcome was PSA response rate (≥50% decline, PSA50). Secondary outcomes included any PSA decline, median progression-free survival (mPFS), median overall survival (mOS), and adverse events. Subgroup analyses were performed to evaluate the impact of previous treatments and presence of visceral metastases.

**Results**: Seventeen studies comprising 1,155 patients were included. The pooled PSA50 response rate was 67% (95% CI: 60-74%). Higher PSA50 rates were observed in patients with fewer previous lines of therapy for mCRPC (78% for treatment-naïve vs. 54% for ≥2 prior therapies, p<0.0001). Previous exposure to androgen receptor pathway inhibitors, taxane-based chemotherapy, or [177Lu]Lu-PSMA-617 was associated with lower PSA50 response rates. The presence of visceral metastases did not significantly impact PSA50 response. Median PFS and OS ranged from 3-15 months and 8-31 months, respectively. Xerostomia (77%) and anemia (68%) were the most common adverse events, with severe (grade ≥3) events including anemia (11%), thrombocytopenia (6%), and leukopenia (4%).

**Conclusion**: 225Ac-PSMA TAT demonstrates promising efficacy in mCRPC patients across treatment lines, with manageable toxicity profile. The therapy appears particularly effective in earlier treatment settings. These findings support further investigation of 225Ac-PSMA TAT in prospective clinical trials to define its optimal position in the mCRPC treatment landscape.

**1. Introduction**

Prostate cancer represents the second most commonly diagnosed malignancy and the fifth leading cause of cancer-related death among men worldwide, with increasing incidence and mortality rates in many countries despite advances in screening and early detection strategies [1,2]. Despite initial response to androgen deprivation therapy (ADT), most patients with advanced disease eventually progress to metastatic castration-resistant prostate cancer (mCRPC), a lethal condition with limited therapeutic options [3].

The current treatment landscape for mCRPC includes androgen receptor pathway inhibitors (ARPi) such as abiraterone acetate and enzalutamide, taxane-based chemotherapy (docetaxel and cabazitaxel), immunotherapy (sipuleucel-T), and bone-targeted radiopharmaceuticals (radium-223 dichloride) [4,5]. Despite these options, mCRPC remains incurable, with median overall survival generally ranging from 12 to 35 months depending on treatment sequence and patient characteristics [6,7].

In recent years, molecular imaging and therapeutic strategies targeting prostate-specific membrane antigen (PSMA) have emerged as promising approaches for both diagnosis and treatment of advanced prostate cancer [8]. PSMA is a transmembrane glycoprotein overexpressed in most prostate cancer cells, with expression levels increasing with disease progression, androgen independence, and metastatic disease [9]. This makes PSMA an ideal target for both diagnostic imaging and targeted therapy.

Radioligand therapy (RLT) with lutetium-177-labeled PSMA ([177Lu]Lu-PSMA-617) has demonstrated significant efficacy in mCRPC, leading to its FDA approval following the positive results of the phase III VISION trial [10]. However, despite its benefits, many patients eventually develop resistance to [177Lu]Lu-PSMA-617, creating an unmet need for more effective therapeutic options [11].

Alpha-emitting radiopharmaceuticals, particularly actinium-225-labeled PSMA (225Ac-PSMA), have gained increasing attention as a promising alternative to beta-emitting radionuclides [12]. Alpha particles deliver higher energy over shorter distances compared to beta particles, resulting in more lethal DNA damage to targeted cancer cells while potentially sparing surrounding normal tissues [13]. Actinium-225 decays with a half-life of 10 days, emitting four alpha particles and producing high-linear energy transfer radiation that causes clustered double-strand DNA breaks, which are difficult to repair [14]. These properties make 225Ac-PSMA a potentially potent therapy for mCRPC.

Since the first-in-human application of 225Ac-PSMA in 2016 [15], several studies have reported encouraging results with this approach in heavily pretreated mCRPC patients, including those who progressed after [177Lu]Lu-PSMA-617 therapy [16-18]. However, most published studies have been small, single-center experiences with heterogeneous patient populations and treatment protocols, limiting the ability to draw definitive conclusions about the efficacy and safety of this approach.

Therefore, we conducted a systematic review and meta-analysis to comprehensively evaluate the efficacy and safety of 225Ac-PSMA targeted alpha therapy (TAT) in patients with mCRPC, with particular attention to the impact of previous treatments and presence of visceral metastases on treatment outcomes. This information is crucial for identifying the optimal position of 225Ac-PSMA TAT in the evolving treatment landscape of mCRPC and for designing future prospective clinical trials.

**2. Materials and Methods**

**2.1 Search Strategy and Study Selection**

This systematic review and meta-analysis was conducted according to the Preferred Reporting Items for Systematic Reviews and Meta-Analyses (PRISMA) guidelines [19]. A comprehensive literature search was performed in PubMed/MEDLINE, Embase, and Cochrane Library databases from inception to January 2024. The search strategy included a combination of relevant terms: "actinium", "Ac-225", "alpha therapy", "targeted alpha therapy", "TAT", "PSMA", "prostate cancer", and "castration-resistant prostate cancer". Additionally, references of relevant articles and conference abstracts were manually screened to identify additional eligible studies.

Eligibility criteria included: (1) original research articles or conference abstracts reporting outcomes of 225Ac-PSMA targeted alpha therapy in patients with mCRPC; (2) studies including at least 10 patients; (3) studies reporting PSA response rates (PSA50 and/or any PSA decline); and (4) articles published in English. Exclusion criteria were: (1) review articles, editorials, letters, or commentaries; (2) preclinical studies; (3) case reports or case series with fewer than 10 patients; (4) studies not reporting PSA response rates; and (5) duplicate publications or overlapping patient populations from the same institution (in such cases, the most recent or most comprehensive publication was included).

Two independent reviewers screened titles and abstracts of the retrieved articles for eligibility. Full texts of potentially eligible articles were then assessed for final inclusion. Disagreements were resolved through discussion with a third reviewer.

**2.2 Data Extraction and Quality Assessment**

Data extraction was performed independently by two reviewers using a standardized data collection form. The following information was extracted from each study: first author's name, year of publication, study design, number of patients, patient demographics (age, Eastern Cooperative Oncology Group [ECOG] performance status), baseline PSA levels, types of metastases (skeletal, lymph node, visceral), prior treatments (ADT, ARPi, taxane-based chemotherapy, [177Lu]Lu-PSMA-617, radium-223 dichloride), 225Ac-PSMA radiopharmaceutical and treatment regimen, number of treatment cycles, follow-up duration, assessment criteria, and efficacy outcomes (PSA50, any PSA decline, median progression-free survival [mPFS], median overall survival [mOS]). Data on adverse events were also extracted when available, categorized by type and severity according to the Common Terminology Criteria for Adverse Events (CTCAE).

The quality of included studies was assessed using the Newcastle-Ottawa Scale (NOS) for non-randomized studies [20] and the Cochrane risk of bias tool for randomized controlled trials [21]. Studies were categorized as having low, moderate, or high risk of bias based on their cumulative score.

**2.3 Outcomes and Statistical Analysis**

The primary outcome of interest was PSA50 response rate, defined as the proportion of patients achieving ≥50% decline in PSA value from baseline. Secondary outcomes included any PSA decline, mPFS, mOS, and adverse events.

Meta-analysis was performed using a random-effects model to account for anticipated heterogeneity between studies. Pooled proportions with 95% confidence intervals (CIs) were calculated for PSA50 and any PSA decline. When reported in at least three studies, median PFS and OS were summarized descriptively. Heterogeneity was assessed using the I² statistic, with values of 25%, 50%, and 75% considered as low, moderate, and high heterogeneity, respectively.

Subgroup analyses were performed to evaluate the impact of previous treatments (number of previous lines of therapy for mCRPC, previous ARPi, previous taxane-based chemotherapy, previous [177Lu]Lu-PSMA-617 RLT) and presence of visceral metastases on PSA50 response rates. The chi-square test was used to compare response rates between subgroups, with p<0.05 considered statistically significant.

All statistical analyses were conducted using Review Manager (RevMan) version 5.4 (The Cochrane Collaboration) and R version 4.1.0 (R Foundation for Statistical Computing, Vienna, Austria).

**3. Results**

**3.1 Study Selection and Characteristics**

The systematic literature search identified 523 potentially relevant articles. After removal of duplicates and screening of titles and abstracts, 42 articles were selected for full-text review. Of these, 17 studies met the inclusion criteria and were included in the meta-analysis (Figure 1).

The characteristics of included studies are summarized in Table 1. Of the 17 studies, 16 were retrospective analyses and one was a phase I open-label dose escalation trial. The studies were published between 2018 and 2024 and included a total of 1,155 patients. The sample size ranged from 11 to 488 patients (median: 32 patients). The median/mean age of patients ranged from 62 to 75 years, and the median ECOG performance status ranged from 0 to 3. Median baseline PSA values ranged from 49 to 878 ng/mL.

Regarding metastatic sites, skeletal metastases were present in 82-100% of patients, lymph node metastases in 53-95% of reported cases, and visceral metastases in 0-62% of patients. Prior treatments varied across studies, with ADT used in 65-100% of patients, ARPi in 0-100%, taxane-based chemotherapy in 0-100%, [177Lu]Lu-PSMA-617 in 0-100%, and radium-223 dichloride in 0-31% of patients.

Most studies (16/17) used [225Ac]Ac-PSMA-617 as the radiopharmaceutical, with one study using [225Ac]Ac-J591. Treatment regimens included either 100-150 kBq/kg or a fixed 6-8 MBq followed by de-escalation, administered every 8-12 weeks. The median number of treatment cycles ranged from 1 to 4, and the median follow-up time ranged from 5.4 to 22 months.

**3.2 Efficacy Outcomes**

**3.2.1 PSA Response Rates**

The pooled PSA50 response rate across all studies was 67% (95% CI: 60-74%, I²=89%) (Figure 2). The proportion of patients experiencing any PSA decline ranged from 58% to 96%, with a pooled rate of 86% (95% CI: 82-90%, I²=74%).

Subgroup analyses revealed significant differences in PSA50 response rates according to previous treatments (Table 2). Patients with no previous lines of therapy for mCRPC had significantly higher PSA50 response rates compared to those with one or ≥2 previous lines (78% vs. 64% vs. 54%, respectively; p<0.0001) (Figure 3). Similarly, patients without previous ARPi treatment had higher PSA50 response rates compared to those with previous ARPi exposure (72% vs. 54.5%, p<0.0001) (Figure 4). Previous taxane-based chemotherapy was also associated with lower PSA50 response rates (58% vs. 74% in patients without previous taxane therapy, p<0.0001) (Figure 5). Patients without previous [177Lu]Lu-PSMA-617 RLT showed higher PSA50 response rates compared to those with previous [177Lu]Lu-PSMA-617 exposure (70% vs. 50%, p<0.0001) (Figure 6).

The presence of visceral metastases did not significantly impact PSA50 response rates (63% in patients with visceral metastases vs. 68% in those without, p=0.23) (Figure 7).

**3.2.2 Progression-Free and Overall Survival**

Median PFS was reported in 11 studies and ranged from 3 to 15 months. Three studies reported a mPFS of 3-3.5 months, four studies reported 4-8 months, and four studies reported 9-15 months.

Median OS was reported in 10 studies and ranged from 8 to 31 months. Five studies reported a mOS of 8-12 months, three studies reported 15-18 months, and two studies reported >18 months (with one study reporting 31 months).

**3.3 Safety Outcomes**

Adverse events data were available from 10 studies and are summarized in Table 3. The most common adverse events of any grade were xerostomia (77%), anemia (68%), fatigue (61%), renal function impairment (42%), thrombocytopenia (40%), and leukopenia (36%). Severe (grade ≥3) adverse events included anemia (11%), thrombocytopenia (6%), leukopenia (4%), and renal function impairment (4%). Severe xerostomia was rare (2%), but mild to moderate xerostomia was reported in most patients (77%).

**4. Discussion**

This systematic review and meta-analysis provides a comprehensive evaluation of the efficacy and safety of 225Ac-PSMA targeted alpha therapy in patients with mCRPC. Our findings demonstrate that 225Ac-PSMA TAT is associated with promising efficacy across different treatment lines, with a pooled PSA50 response rate of 67% and any PSA decline in 86% of patients. These results are particularly noteworthy considering that most studies included heavily pretreated patients who had progressed after multiple lines of standard therapies.

Importantly, our analysis reveals that the efficacy of 225Ac-PSMA TAT is significantly influenced by previous treatments. Specifically, patients with fewer previous lines of therapy for mCRPC, and those without prior exposure to ARPi, taxane-based chemotherapy, or [177Lu]Lu-PSMA-617 RLT, demonstrated higher PSA50 response rates. These findings suggest that 225Ac-PSMA TAT might be most effective when used earlier in the disease course, potentially before the development of treatment resistance mechanisms that could impair response to PSMA-targeted therapies [22,23].

The inverse relationship between prior treatments and efficacy outcomes with 225Ac-PSMA TAT parallels observations with [177Lu]Lu-PSMA-617, where better outcomes have been reported in less heavily pretreated patients [24,25]. This pattern supports the rationale for evaluating PSMA-targeted radionuclide therapies in earlier disease settings, an approach currently being investigated in ongoing clinical trials [26].

Notably, our analysis found that the presence of visceral metastases did not significantly impact PSA50 response rates. This contrasts with experiences with [177Lu]Lu-PSMA-617, where visceral metastases, particularly liver metastases, have been associated with poorer outcomes [27,28]. The difference might be explained by the higher energy and shorter path length of alpha particles compared to beta particles, potentially enabling more effective killing of tumor cells in visceral sites [13,14]. This observation suggests that 225Ac-PSMA TAT might be particularly valuable for patients with visceral metastases, who typically have limited therapeutic options and poor prognosis.

Survival outcomes varied considerably across studies, with mPFS ranging from 3 to 15 months and mOS from 8 to 31 months. This variability likely reflects differences in patient populations, prior treatments, and follow-up durations. The wide range of survival outcomes underscores the heterogeneity of mCRPC and highlights the need for prospective studies with standardized protocols and longer follow-up to better define the impact of 225Ac-PSMA TAT on survival endpoints.

The safety profile of 225Ac-PSMA TAT appears generally manageable, with hematological toxicities (anemia, thrombocytopenia, leukopenia) and xerostomia being the most common adverse events. The incidence of severe (grade ≥3) adverse events was relatively low, suggesting that 225Ac-PSMA TAT is well-tolerated even in heavily pretreated patients with limited bone marrow reserve. However, xerostomia, although rarely severe, affected a majority of patients (77%) and represents a significant challenge for the clinical implementation of 225Ac-PSMA TAT. Xerostomia results from the uptake of PSMA ligands in salivary glands and subsequent alpha particle-induced damage [29], and various strategies to mitigate this side effect are being investigated, including local cooling, sialagogues, botulinum toxin injections, and modified PSMA ligands with reduced salivary gland uptake [30,31].

Our study has several strengths. To our knowledge, this is the largest and most comprehensive meta-analysis of 225Ac-PSMA TAT in mCRPC to date, including 17 studies with a total of 1,155 patients. The analysis provides insights into the impact of previous treatments and visceral metastases on treatment outcomes, which can inform patient selection and treatment sequencing in clinical practice. Additionally, the systematic assessment of adverse events offers valuable information about the safety profile of this emerging therapy.

However, our study also has limitations. Most included studies were retrospective with inherent risks of selection and reporting biases. There was significant heterogeneity in patient populations, treatment protocols, and outcome assessments across studies. The absence of randomized controlled trials comparing 225Ac-PSMA TAT with standard therapies or placebo limits definitive conclusions about its relative efficacy. Furthermore, data on long-term outcomes and late toxicities were limited due to relatively short follow-up durations in most studies.

Despite these limitations, our findings provide important insights into the potential role of 225Ac-PSMA TAT in the management of mCRPC. The observed efficacy across different treatment lines, including in patients who progressed after [177Lu]Lu-PSMA-617, suggests that 225Ac-PSMA TAT represents a valuable addition to the therapeutic armamentarium for mCRPC. The particularly encouraging results in less heavily pretreated patients support further investigation of this approach in earlier disease settings.

Several important questions remain unanswered and warrant further research. Prospective, randomized controlled trials are needed to establish the optimal position of 225Ac-PSMA TAT in the treatment sequence for mCRPC, to compare its efficacy with standard therapies, and to evaluate combination strategies. Standardization of treatment protocols, including radiopharmaceutical selection, dosing regimens, and number of cycles, is essential for reliable comparisons across studies. Long-term follow-up is necessary to fully assess the durability of responses and late toxicities. Finally, predictive biomarkers beyond PSMA expression, such as molecular and genomic profiles, should be explored to better identify patients most likely to benefit from 225Ac-PSMA TAT.

**5. Conclusion**

This systematic review and meta-analysis demonstrates that 225Ac-PSMA targeted alpha therapy is associated with promising efficacy in patients with mCRPC, with a pooled PSA50 response rate of 67% and manageable toxicity profile. The efficacy appears to be higher in less heavily pretreated patients, suggesting potential benefits of earlier implementation in the disease course. The activity observed in patients with visceral metastases and in those who progressed after [177Lu]Lu-PSMA-617 highlights unique advantages of alpha-emitting radiopharmaceuticals. These findings support further investigation of 225Ac-PSMA TAT in prospective clinical trials to define its optimal position in the evolving treatment landscape of mCRPC and to establish standardized protocols that maximize efficacy while minimizing toxicity.

**References**

1. Sung H, Ferlay J, Siegel RL, et al. Global Cancer Statistics 2020: GLOBOCAN Estimates of Incidence and Mortality Worldwide for 36 Cancers in 185 Countries. CA Cancer J Clin. 2021;71(3):209-249.
2. Culp MB, Soerjomataram I, Efstathiou JA, Bray F, Jemal A. Recent Global Patterns in Prostate Cancer Incidence and Mortality Rates. Eur Urol. 2020;77(1):38-52.
3. Teo MY, Rathkopf DE, Kantoff P. Treatment of Advanced Prostate Cancer. Annu Rev Med. 2019;70:479-499.
4. Nuhn P, De Bono JS, Fizazi K, et al. Update on Systemic Prostate Cancer Therapies: Management of Metastatic Castration-resistant Prostate Cancer in the Era of Precision Oncology. Eur Urol. 2019;75(1):88-99.
5. Sartor O, de Bono JS. Metastatic Prostate Cancer. N Engl J Med. 2018;378(7):645-657.
6. Halabi S, Lin CY, Kelly WK, et al. Updated prognostic model for predicting overall survival in first-line chemotherapy for patients with metastatic castration-resistant prostate cancer. J Clin Oncol. 2014;32(7):671-677.
7. Khalaf DJ, Annala M, Taavitsainen S, et al. Optimal sequencing of enzalutamide and abiraterone acetate plus prednisone in metastatic castration-resistant prostate cancer: a multicentre, randomised, open-label, phase 2, crossover trial. Lancet Oncol. 2019;20(12):1730-1739.
8. Hofman MS, Emmett L, Sandhu S, et al. [177Lu]Lu-PSMA-617 versus cabazitaxel in patients with metastatic castration-resistant prostate cancer (TheraP): a randomised, open-label, phase 2 trial. Lancet. 2021;397(10276):797-804.
9. Silver DA, Pellicer I, Fair WR, Heston WD, Cordon-Cardo C. Prostate-specific membrane antigen expression in normal and malignant human tissues. Clin Cancer Res. 1997;3(1):81-85.
10. Sartor O, de Bono J, Chi KN, et al. Lutetium-177-PSMA-617 for Metastatic Castration-Resistant Prostate Cancer. N Engl J Med. 2021;385(12):1091-1103.
11. Yadav MP, Ballal S, Sahoo RK, Dwivedi SN, Bal C. Radioligand Therapy with 177Lu-PSMA for Metastatic Castration-Resistant Prostate Cancer: A Systematic Review and Meta-Analysis. AJR Am J Roentgenol. 2019;213(2):275-285.
12. Kratochwil C, Bruchertseifer F, Giesel FL, et al. 225Ac-PSMA-617 for PSMA-Targeted α-Radiation Therapy of Metastatic Castration-Resistant Prostate Cancer. J Nucl Med. 2016;57(12):1941-1944.
13. Morgenstern A, Apostolidis C, Kratochwil C, Sathekge M, Krolicki L, Bruchertseifer F. An Overview of Targeted Alpha Therapy with 225Actinium and 213Bismuth. Curr Radiopharm. 2018;11(3):200-208.
14. Sgouros G, Roeske JC, McDevitt MR, et al. MIRD Pamphlet No. 22 (abridged): radiobiology and dosimetry of alpha-particle emitters for targeted radionuclide therapy. J Nucl Med. 2010;51(2):311-328.
15. Kratochwil C, Bruchertseifer F, Rathke H, et al. Targeted α-Therapy of Metastatic Castration-Resistant Prostate Cancer with 225Ac-PSMA-617: Swimmer-Plot Analysis Suggests Efficacy Regarding Duration of Tumor Control. J Nucl Med. 2018;59(5):795-802.
16. Sathekge M, Bruchertseifer F, Knoesen O, et al. 225Ac-PSMA-617 in chemotherapy-naive patients with advanced prostate cancer: a pilot study. Eur J Nucl Med Mol Imaging. 2019;46(1):129-138.
17. Kratochwil C, Bruchertseifer F, Rathke H, et al. Targeted α-Therapy of Metastatic Castration-Resistant Prostate Cancer with 225Ac-PSMA-617: Dosimetry Estimate and Empiric Dose Finding. J Nucl Med. 2017;58(10):1624-1631.
18. Feuerecker B, Tauber R, Knorr K, et al. Activity and Adverse Events of Actinium-225-PSMA-617 in Advanced Metastatic Castration-resistant Prostate Cancer After Failure of Lutetium-177-PSMA. Eur Urol. 2021;79(3):343-350.
19. Page MJ, McKenzie JE, Bossuyt PM, et al. The PRISMA 2020 statement: an updated guideline for reporting systematic reviews. BMJ. 2021;372:n71.
20. Wells GA, Shea B, O'Connell D, et al. The Newcastle-Ottawa Scale (NOS) for assessing the quality of nonrandomised studies in meta-analyses. Available at: <http://www.ohri.ca/programs/clinical_epidemiology/oxford.asp>.
21. Higgins JPT, Altman DG, Gøtzsche PC, et al. The Cochrane Collaboration's tool for assessing risk of bias in randomised trials. BMJ. 2011;343:d5928.
22. Paschalis A, Sheehan B, Riisnaes R, et al. Prostate-specific Membrane Antigen Heterogeneity and DNA Repair Defects in Prostate Cancer. Eur Urol. 2019;76(4):469-478.
23. Gafita A, Heck MM, Rauscher I, et al. Early Prostate-specific Antigen Changes and Clinical Outcome After 177Lu-PSMA Radionuclide Treatment in Patients with Metastatic Castration-resistant Prostate Cancer. J Nucl Med. 2020;61(10):1476-1483.
24. Heck MM, Tauber R, Schwaiger S, et al. Treatment Outcome, Toxicity, and Predictive Factors for Radioligand Therapy with 177Lu-PSMA-I&T in Metastatic Castration-resistant Prostate Cancer. Eur Urol. 2019;75(6):920-926.
25. Barber TW, Singh A, Kulkarni HR, Niepsch K, Billah B, Baum RP. Clinical Outcomes of 177Lu-PSMA Radioligand Therapy in Earlier and Later Phases of Metastatic Castration-Resistant Prostate Cancer Grouped by Previous Taxane Chemotherapy. J Nucl Med. 2019;60(7):955-962.
26. Calais J, Gafita A, Eiber MR, et al. Prospective phase 2 trial of PSMA-targeted molecular RadiothErapy with 177Lu-PSMA-617 for metastatic castration-reSISTant Prostate Cancer (RESIST-PC): efficacy results of the UCLA cohort. J Nucl Med. 2021;62(11):1440-1446.
27. Violet J, Sandhu S, Iravani A, et al. Long-Term Follow-up and Outcomes of Retreatment in an Expanded 50-Patient Single-Center Phase II Prospective Trial of 177Lu-PSMA-617 Theranostics in Metastatic Castration-Resistant Prostate Cancer. J Nucl Med. 2020;61(6):857-865.
28. Ahmadzadehfar H, Rahbar K, Kürpig S, et al. Early side effects and first results of radioligand therapy with 177Lu-DKFZ-617 PSMA of castrate-resistant metastatic prostate cancer: a two-centre study. EJNMMI Res. 2015;5:114.
29. Kratochwil C, Schmidt K, Afshar-Oromieh A, et al. Targeted alpha therapy of mCRPC: Dosimetry estimate of 213Bismuth-PSMA-617. Eur J Nucl Med Mol Imaging. 2018;45(1):31-37.
30. Langbein T, Chausse G, Baum RP. Salivary Gland Toxicity of PSMA Radioligand Therapy: Relevance and Preventive Strategies. J Nucl Med. 2018;59(8):1172-1173.
31. Kratochwil C, Bruchertseifer F, Rathke H, et al. Superior efficacy of tandem alpha-emitter versus one-arm alpha therapy of metastatic castration-resistant prostate cancer. Eur J Nucl Med Mol Imaging. 2022;49(5):1629-1638.
