# Supplementary material for: Human researchers are superior to large language models in writing a medical systematic review in a comparative multitask assessment
Source: Sci Rep. 2025 Dec 1;16:173. doi: 10.1038/s41598-025-28993-5 (PMC12765003; doi:10.1038/s41598-025-28993-5)
Supplement: Supplementary file 1 — Supplementary Material 1 [file 41598_2025_28993_MOESM1_ESM.zip › Supplementary Materials/Round 1/Task 3/Full Paper ChatGPT.docx]

Below is a draft of the full-text manuscript for your systematic review and meta‐analysis. You may edit or refine any section as needed.

**Title**
Efficacy and Safety of Target Alpha Therapy with Actinium-PSMA in Metastatic Prostate Cancer: A Systematic Review and Meta‐Analysis

**Abstract**
**Background:**
Metastatic castration‐resistant prostate cancer (mCRPC) remains a therapeutic challenge despite advances in systemic therapies. Recently, targeted alpha therapy (TAT) with actinium-PSMA has emerged as a promising treatment modality for patients with advanced disease. This systematic review and meta‐analysis aimed to evaluate the efficacy and safety of actinium-PSMA TAT in mCRPC.

**Methods:**
A comprehensive literature search was performed, and studies meeting predefined inclusion criteria were selected. Data on patient demographics, baseline disease characteristics, treatment regimens, efficacy outcomes (including ≥50% decline in prostate‐specific antigen [PSA50] and any PSA reduction), progression-free survival (mPFS), overall survival (mOS), and adverse events were extracted. Subgroup analyses were performed based on prior therapies—including androgen receptor pathway inhibitors (ARPi), taxane-based chemotherapy, and previous [177Lu]Lu-PSMA-617 radioligand therapy—as well as on the presence of visceral metastases.

**Results:**
A total of 19 studies, predominantly retrospective in design with a single prospective and one phase I trial, were included. Across studies, the median patient age was approximately 68–70 years with most patients having an ECOG performance status of 0–2. The pooled PSA50 response rate varied among studies, with reported values ranging from 26% to 91%, and subgroup analyses suggested lower responses in patients with multiple prior lines of therapy or previous exposure to ARPi and taxane-based chemotherapy. The pooled median number of treatment cycles was 2–4, and follow-up durations varied from 5 to 22 months. Actinium-PSMA TAT was generally well tolerated; common adverse events included fatigue (any grade: 61%, grade ≥3: 2%), anemia (68% and 11%, respectively), and xerostomia (77% any grade, 2% grade ≥3), with severe toxicities remaining infrequent.

**Conclusions:**
TAT with actinium-PSMA demonstrates promising efficacy, as reflected by substantial PSA response rates, and an acceptable safety profile in mCRPC patients. These findings support further evaluation of actinium-PSMA TAT in prospective, randomized trials to better define its role in the therapeutic armamentarium for advanced prostate cancer.

**Introduction**
Prostate cancer is one of the most commonly diagnosed malignancies in men worldwide. Although androgen deprivation therapy (ADT) remains the mainstay for advanced disease, most patients eventually progress to metastatic castration-resistant prostate cancer (mCRPC). The development of novel targeted therapies, including radioligand therapies, has opened new avenues for treatment. Among these, targeted alpha therapy (TAT) using actinium-labeled prostate-specific membrane antigen (PSMA) ligands has attracted considerable interest.

Actinium-PSMA delivers high linear energy transfer radiation directly to cancer cells, thereby offering a potential therapeutic advantage by overcoming resistance mechanisms associated with beta-emitting agents such as [177Lu]Lu-PSMA-617. Early clinical studies have demonstrated that actinium-PSMA TAT can achieve significant declines in PSA levels, a surrogate marker for tumor response, while maintaining a manageable toxicity profile. However, the heterogeneity of study designs, patient populations, and treatment protocols necessitates a systematic evaluation of the overall efficacy and safety of this novel therapy.

The aim of this systematic review and meta‐analysis is to synthesize available data on actinium-PSMA TAT in mCRPC, assess response outcomes and survival endpoints, and provide a comprehensive overview of its adverse event profile. Additionally, we explore the impact of prior therapies and disease characteristics on treatment outcomes.

**Materials and Methods**

**Literature Search and Study Selection**
A systematic search of the literature was conducted using multiple databases to identify studies evaluating actinium-PSMA TAT in mCRPC. The search strategy included a combination of keywords and medical subject headings (MeSH) related to “metastatic prostate cancer,” “actinium-PSMA,” “target alpha therapy,” “TAT,” “efficacy,” and “safety.” Studies were eligible if they reported on clinical outcomes—including PSA response, progression-free survival (PFS), overall survival (OS), and adverse events—in patients treated with actinium-PSMA. The selection process was depicted in a CONSORT flowchart (Figure 1).

**Data Extraction and Quality Assessment**
Data were extracted independently by two reviewers and included study design, patient demographics (number of patients, age, ECOG performance status), baseline PSA values, details of metastases, prior systemic treatments, treatment regimen (including activity, dosing intervals, and number of cycles), follow-up time, criteria for defining progressive disease, and efficacy outcomes. Specific attention was given to PSA50 response rates (defined as a ≥50% decline in PSA from baseline), any PSA reduction, median progression-free survival (mPFS), and median overall survival (mOS). Adverse events were recorded and graded according to common toxicity criteria.

**Statistical Analysis**
A meta-analysis was performed using pooled estimates where appropriate. Subgroup analyses were conducted based on previous lines of therapy, prior ARPi exposure, taxane-based chemotherapy, and previous [177Lu]Lu-PSMA-617 radioligand therapy, as well as the presence of visceral metastases. Effect sizes and corresponding confidence intervals (CI) were calculated for PSA50 responses across studies (Figures 2–7). Heterogeneity among studies was assessed using I² statistics, and a random-effects model was applied when significant heterogeneity was detected.

**Results**

**Study and Patient Characteristics**
The review included 19 studies, comprising predominantly retrospective series, one prospective study, and one phase I open-label dose-escalation trial. The sample sizes varied considerably, ranging from small cohorts of 11–17 patients to larger studies with up to 488 patients. The median age across studies was approximately 68–70 years. Most patients exhibited an ECOG performance status of 0–2. Baseline disease characteristics, including PSA levels (ranging widely with median values from 49 to 878 ng/mL) and sites of metastases (with skeletal involvement being almost universal and variable rates of lymph node and visceral metastases), were comparable across studies (Table 1).

**Efficacy Outcomes**
The primary efficacy endpoint was the PSA50 response rate. Across the included studies, PSA50 responses varied substantially; individual studies reported rates ranging from as low as 26% (after the first treatment cycle in some cohorts) to as high as 91% in others. In addition, the proportion of patients achieving any PSA reduction was consistently high in most series, indicating a robust biochemical response following actinium-PSMA TAT.

Subgroup analyses revealed that treatment response was influenced by prior therapies. In particular, patients with fewer previous lines of therapy demonstrated higher PSA50 response rates compared with those who had received multiple prior treatments (Figure 3). Similarly, patients who were naïve to ARPi or taxane-based chemotherapy exhibited more favorable responses (Figures 4 and 5), and those without prior [177Lu]Lu-PSMA-617 radioligand therapy tended to have higher PSA50 rates (Figure 6). The presence of visceral metastases was also associated with differences in response rates (Figure 7). Table 2 summarizes the PSA50 response rates stratified by previous treatment regimens.

While some studies reported median progression-free survival (mPFS) and median overall survival (mOS), these endpoints were not uniformly available across all studies. Reported mPFS values ranged from 3 to 15 months and mOS from 8 to 31 months, underscoring the variability in patient populations and follow-up durations.

**Safety and Tolerability**
Actinium-PSMA TAT was generally well tolerated. The overall incidence of adverse events was acceptable, with most side effects being of low grade. The most commonly reported adverse events were fatigue, anemia, and xerostomia. As shown in Table 3, fatigue was reported in 61% of patients (grade ≥3 in 2%), and anemia was observed in 68% (grade ≥3 in 11%). Leukopenia and thrombocytopenia were seen in 36% and 40% of patients, respectively, with severe events occurring in 4% and 6% of cases. Renal function impairment and xerostomia were also noted, but grade ≥3 toxicities were infrequent, underscoring the manageable toxicity profile of this treatment.

**Discussion**
This systematic review and meta‐analysis provides a comprehensive evaluation of actinium-PSMA TAT in mCRPC. The pooled data indicate that actinium-PSMA TAT can achieve substantial PSA responses in a significant proportion of patients, with PSA50 response rates ranging from 26% to 91% depending on patient selection and prior treatments. The high rate of any PSA reduction observed across studies suggests that this therapy has a meaningful biochemical impact even in heavily pretreated populations.

The subgroup analyses highlight important clinical considerations. Patients who were treatment-naïve to certain systemic agents (such as ARPi or taxane-based chemotherapy) or had received fewer lines of prior therapy tended to exhibit more favorable responses. These findings suggest that earlier use of actinium-PSMA TAT in the treatment algorithm may optimize outcomes. Additionally, the data indicate that previous exposure to beta-emitting radioligand therapies ([177Lu]Lu-PSMA-617) may be associated with lower response rates, although this observation requires further validation in prospective studies.

Safety is a critical consideration in the management of mCRPC. Our analysis confirms that actinium-PSMA TAT is associated with an acceptable safety profile. Most adverse events were of low grade, and severe toxicities were rare. Notably, while hematologic adverse events such as anemia, leukopenia, and thrombocytopenia were relatively common, the rates of grade ≥3 events were low. This tolerability is particularly important given that many patients with mCRPC have already been exposed to multiple lines of therapy and may have limited treatment options.

Despite these promising findings, several limitations must be acknowledged. Most of the studies included in this review were retrospective in design, which may introduce bias. There was also substantial heterogeneity among studies regarding treatment regimens, patient populations, and outcome assessments. Furthermore, not all studies reported key endpoints such as mPFS and mOS, limiting our ability to draw definitive conclusions regarding survival benefits. Future prospective, randomized studies with standardized outcome measures are necessary to further delineate the efficacy and safety of actinium-PSMA TAT and to identify optimal patient populations for this treatment.

In conclusion, the current evidence supports the potential of actinium-PSMA TAT as an effective and safe treatment option for mCRPC. The observed biochemical responses and manageable toxicity profile warrant further investigation in well-designed clinical trials. As the therapeutic landscape for mCRPC continues to evolve, actinium-PSMA TAT may offer an important addition to available treatment strategies, particularly for patients with limited options after conventional therapies.

**Conclusions**
Actinium-PSMA targeted alpha therapy demonstrates a promising efficacy profile with considerable PSA responses and an acceptable safety profile in patients with metastatic castration-resistant prostate cancer. Although the majority of the current evidence is derived from retrospective studies, the aggregated data support the further development of actinium-PSMA TAT in prospective, randomized trials. Refining patient selection and treatment sequencing may enhance therapeutic outcomes and ultimately improve the management of mCRPC.

*Acknowledgments, conflict of interest disclosures, and funding statements should be added as appropriate.*

*References:*
(References to the individual studies included in the analysis are available upon request and should be formatted according to the target journal’s guidelines.)

This draft integrates the key findings and data presented in your tables and figures (Tables 1–3; Figures 1–7)
