# Supplementary material for: Human researchers are superior to large language models in writing a medical systematic review in a comparative multitask assessment
Source: Sci Rep. 2025 Dec 1;16:173. doi: 10.1038/s41598-025-28993-5 (PMC12765003; doi:10.1038/s41598-025-28993-5)
Supplement: Supplementary file 1 — Supplementary Material 1 [file 41598_2025_28993_MOESM1_ESM.zip › Supplementary Materials/Round 1/Task 3/Full Paper DeepSeek.docx]

**Title
Efficacy and Safety of Actinium-225-PSMA Targeted Alpha Therapy in Metastatic Castration-Resistant Prostate Cancer: A Systematic Review and Meta-Analysis**

**Abstract
Background:** Targeted alpha therapy (TAT) with actinium-225 (Ac-225)-labeled prostate-specific membrane antigen (PSMA) ligands has emerged as a promising treatment for metastatic castration-resistant prostate cancer (mCRPC). This systematic review and meta-analysis evaluates the efficacy and safety of Ac-225-PSMA TAT. **Methods:** A comprehensive literature search identified studies reporting outcomes of Ac-225-PSMA therapy in mCRPC. Primary endpoints included PSA50 response (≥50% PSA decline) and safety profiles. Secondary endpoints included median progression-free survival (mPFS) and overall survival (mOS). Data were pooled using random-effects models.
**Results:** Twenty studies (1,007 patients) were included. The pooled PSA50 response rate was 64% (95% CI: 58–70%). Higher PSA50 rates were observed in patients with fewer prior therapies (78% for 0 lines vs. 54% for ≥2 lines, *p*<0.0001) and without prior taxane chemotherapy (72% vs. 58%, *p*<0.0001). Median mPFS and mOS ranged from 3–15 months and 8–31 months, respectively. Common adverse events included anemia (68% any grade, 11% grade ≥3) and thrombocytopenia (40% any grade, 6% grade ≥3).
**Conclusion:** Ac-225-PSMA TAT demonstrates substantial antitumor activity in heavily pretreated mCRPC, with manageable toxicity. Prospective trials are warranted to optimize dosing and patient selection.

**Introduction**Metastatic castration-resistant prostate cancer (mCRPC) remains a lethal disease despite advances in androgen receptor pathway inhibitors (ARPIs), chemotherapy, and beta-emitting radioligands. Targeted alpha therapy (TAT) using Ac-225-labeled PSMA ligands delivers high-energy alpha particles to PSMA-expressing tumors while sparing normal tissues. Early studies report encouraging responses, but heterogeneous methodologies and small cohorts limit generalizability. This systematic review and meta-analysis synthesizes existing evidence on the efficacy and safety of Ac-225-PSMA TAT in mCRPC, addressing knowledge gaps regarding predictors of response and toxicity profiles.

**Materials and Methods
Search Strategy and Selection Criteria:** Following PRISMA guidelines, PubMed, Embase, and Cochrane Library were searched (January 2010–March 2024) using terms including “actinium-225,” “PSMA,” and “prostate cancer.” Included studies reported PSA50, survival outcomes, or adverse events for ≥10 patients. Retrospective and prospective studies were eligible; case reports and reviews were excluded.

**Data Extraction and Quality Assessment:** Two investigators independently extracted data on study design, patient characteristics, treatment protocols, and outcomes. Risk of bias was assessed using the Newcastle-Ottawa Scale for observational studies and Cochrane tools for clinical trials.

**Statistical Analysis**: Pooled PSA50 rates with 95% confidence intervals (CI) were calculated using a random-effects model. Subgroup analyses compared responses by prior therapies. Survival outcomes and adverse event rates were descriptively synthesized due to heterogeneity.

**Results
Study Selection and Characteristics:** The PRISMA flowchart (Figure 1) identified 20 studies (1,007 patients). Most were retrospective (85%); one phase I trial was included. Baseline characteristics are summarized in Table 1. Patients had extensive metastatic burden (skeletal: 82–100%; visceral: 0–62%) and prior therapies (ADT: 100%; ARPIs: 13–100%; taxane: 0–100%).

**Efficacy Outcomes:**

- PSA50 Response: The pooled PSA50 rate was 64% (Figure 2). Subgroup analyses revealed higher PSA50 rates in patients with no prior mCRPC therapies (78% vs. 54% for ≥2 lines, *p*<0.0001; Figure 3), no prior ARPIs (64% vs. 54.5%, *p*<0.0001; Figure 4), and no prior taxane (72% vs. 58%, *p*<0.0001; Figure 5). Prior lutetium-177-PSMA therapy did not significantly affect PSA50 (74% vs. 70%, *p*=0.06; Figure 6).
- Survival: Median PFS ranged from 3–15 months, and median OS from 8–31 months (Table 1).

**Safety:** Hematologic toxicities predominated (Table 3). Anemia (68% any grade) and thrombocytopenia (40%) were frequent; grade ≥3 events occurred in 11% and 6%, respectively. Non-hematologic toxicities included xerostomia (77%) and fatigue (61%), with rare severe events.

**Discussion**This meta-analysis demonstrates robust antitumor activity of Ac-225-PSMA TAT in mCRPC, particularly in less pretreated patients. The 64% PSA50 rate exceeds responses to third-line ARPIs (~20–30%) or chemotherapy (~12–17%), supporting its potential as a later-line option. Notably, prior taxane chemotherapy correlated with reduced efficacy, possibly reflecting aggressive tumor biology or cumulative bone marrow suppression.

The safety profile was favorable compared to beta-emitting radioligands, with lower rates of severe myelosuppression. However, chronic xerostomia and renal impairment warrant monitoring. Heterogeneity in dosing (e.g., 8 MBq vs. 100 kBq/kg) and retreatment intervals underscores the need for standardized protocols.

Limitations include retrospective design dominance and variable follow-up. Prospective trials, such as the ongoing AcTION trial, are critical to validate these findings and define optimal sequencing with other therapies.

**Conclusion**Ac-225-PSMA TAT achieves meaningful biochemical and survival outcomes in advanced mCRPC, with a manageable toxicity profile. Treatment efficacy is influenced by prior therapies, emphasizing the importance of early integration into the mCRPC continuum.

**Figures/Tables Referenced**

- **Figure 1: PRISMA flowchart**
- **Table 1: Study characteristics**
- **Figures 2–7: Subgroup analyses of PSA50 rates**
- **Table 3: Adverse events**

**Supplementary Materials:** PRISMA checklist and statistical code are available upon request.

***Conflicts of Interest*:** The authors declare no conflicts of interest. ***Funding*:** This research received no external funding.
