# Supplementary material for: Human researchers are superior to large language models in writing a medical systematic review in a comparative multitask assessment
Source: Sci Rep. 2025 Dec 1;16:173. doi: 10.1038/s41598-025-28993-5 (PMC12765003; doi:10.1038/s41598-025-28993-5)
Supplement: Supplementary file 1 — Supplementary Material 1 [file 41598_2025_28993_MOESM1_ESM.zip › Supplementary Materials/Round 1/Task 3/Full Paper Gemini.docx]

**I now have all the data files needed to complete the scientific paper. Please wait while I generate the full manuscript, incorporating all the information you have provided.**

**Targeted Alpha Therapy with Actinium-PSMA for Metastatic Castration-Resistant Prostate Cancer: A Systematic Review and Meta-Analysis**

**Abstract**

**Background:** Metastatic castration-resistant prostate cancer (mCRPC) remains a significant clinical challenge. Targeted alpha therapy (TAT) with actinium-225 labeled prostate-specific membrane antigen (PSMA) ligands ([<sup>225</sup>Ac]Ac-PSMA) is emerging as a promising treatment. This systematic review and meta-analysis aims to evaluate the efficacy and safety of [<sup>225</sup>Ac]Ac-PSMA TAT in mCRPC.

**Methods:** A systematic literature search was conducted to identify studies evaluating [<sup>225</sup>Ac]Ac-PSMA TAT in mCRPC using PubMed, Web of Science, Scopus, and Embase databases from inception to December 2024. Studies were included if they reported PSA50 response rates in patients with mCRPC treated with [<sup>225</sup>Ac]Ac-PSMA TAT. Data on study design, patient characteristics, treatment regimens, PSA50 response rates (≥50% decline from baseline), and adverse events were extracted. A meta-analysis was performed to pool PSA50 response rates using a random-effects model. Subgroup analyses were conducted based on prior lines of therapy, androgen receptor pathway inhibitor (ARPI) use, taxane-based chemotherapy, lutetium-177-based radioligand therapy (RLT), and presence of visceral metastases.

**Results:** A total of 18 studies with 1007 patients were included in the meta-analysis, as depicted in the CONSORT flowchart. The pooled PSA50 response rate for [<sup>225</sup>Ac]Ac-PSMA TAT in mCRPC was 70% (95% Confidence Interval (CI): 61-79%, p<0.001), with significant heterogeneity (I<sup>2</sup> = 75.95%). Subgroup analyses revealed a PSA50 response rate of 83% (95% CI: 71-93%) in RLT-naïve patients and 54% (95% CI: 43-65%) in RLT-pretreated patients (p<0.001). In ARPI-naïve patients, the PSA50 response rate was 83% (95% CI: 71-93%) compared to 57% (95% CI: 48-65%) in ARPI-pretreated patients (p<0.001). Similarly, PSA50 response rates were 80% (95% CI: 67-91%) in chemotherapy-naïve and 62% (95% CI: 51-72%) in chemotherapy-pretreated patients (p<0.001). Stratification by previous lines of therapy showed PSA50 response rates of 82% (95% CI: 73-90%) in patients with no prior lines of therapy, 68% (95% CI: 57-78%) in patients with one prior line, and 52% (95% CI: 39-65%) in patients with two or more prior lines of therapy for mCRPC (p<0.001). Patients without visceral metastases had a PSA50 response rate of 69% (95% CI: 54-83%), while those with visceral metastases had a response rate of 61% (95% CI: 51-71%), with no significant difference between groups (p=0.276). The most common adverse events of any grade were xerostomia (77%), anemia (68%), fatigue (61%), thrombocytopenia (40%), leukopenia (36%), and renal function impairment (42%). Grade ≥3 adverse events were less frequent, with anemia (11%) being the most common.

**Conclusion:** [<sup>225</sup>Ac]Ac-PSMA TAT demonstrates promising efficacy in mCRPC, with high PSA50 response rates. Efficacy appears to be greater in treatment-naïve settings and decreases with increasing lines of prior therapies, particularly with prior RLT. [<sup>225</sup>Ac]Ac-PSMA TAT is associated with manageable toxicity. Further prospective, randomized controlled trials are warranted to confirm these findings, to better define patient selection criteria, and to optimize the sequencing of [<sup>225</sup>Ac]Ac-PSMA TAT in the treatment landscape of mCRPC.

**Keywords: Prostate cancer, metastatic castration-resistant prostate cancer, target alpha therapy, actinium-225 PSMA, systematic review, meta-analysis, PSA response.**

**1. Introduction**

Prostate cancer is a leading cause of cancer-related mortality in men worldwide. While localized prostate cancer is often curable, a significant proportion of patients develop metastatic disease. Androgen deprivation therapy (ADT) is the cornerstone of treatment for metastatic prostate cancer, but most patients eventually progress to metastatic castration-resistant prostate cancer (mCRPC). Once castration resistance develops, subsequent treatment options, including androgen receptor pathway inhibitors (ARPIs), taxane-based chemotherapy, and radioligand therapy (RLT) with lutetium-177 PSMA ([<sup>177</sup>Lu]Lu-PSMA), offer progressively shorter survival benefits. Therefore, novel therapeutic strategies are urgently needed to improve outcomes for patients with mCRPC.

Targeted alpha therapy (TAT) is an emerging treatment modality in nuclear medicine that utilizes alpha-emitting radionuclides to deliver highly potent and localized radiation to tumor cells while minimizing damage to surrounding healthy tissues. Actinium-225 ([<sup>225</sup>Ac]) is an alpha-emitting radionuclide with a short half-life (10 days) and high linear energy transfer, making it particularly attractive for TAT. Prostate-specific membrane antigen (PSMA) is a well-validated target for imaging and therapy in prostate cancer, as it is highly overexpressed in mCRPC cells. The combination of [<sup>225</sup>Ac]Ac with PSMA-targeting ligands ([<sup>225</sup>Ac]Ac-PSMA) has shown promising preclinical and early clinical activity in mCRPC.

Given the increasing interest in and clinical application of [<sup>225</sup>Ac]Ac-PSMA TAT, a comprehensive evaluation of the available evidence is warranted. This systematic review and meta-analysis aims to assess the efficacy and safety of [<sup>225</sup>Ac]Ac-PSMA TAT in patients with mCRPC by pooling data from published studies, focusing on prostate-specific antigen (PSA) response rates as a primary efficacy endpoint.

**2. Materials and Methods**

**2.1. Search Strategy and Study Selection**

A systematic literature search was conducted in PubMed, Web of Science, Scopus, and Embase databases from inception to December 2024. The search strategy included terms related to "prostate cancer," "metastatic castration-resistant prostate cancer," "target alpha therapy," "actinium-225," and "PSMA." Studies were included if they were original research articles, enrolling patients with mCRPC, evaluating [<sup>225</sup>Ac]Ac-PSMA TAT, and reporting PSA50 response rates. Abstracts, case reports, reviews, and preclinical studies were excluded. Study selection was performed independently by two reviewers ( [*Please insert your initials or names here*] ), with disagreements resolved by consensus. The study selection process is summarized in a CONSORT flowchart (Figure 1).

**2.2. Data Extraction**

Data extraction was performed using a pre-defined data extraction form. Extracted data included: study characteristics (first author, publication year, study design, country), patient demographics (number of patients, age, baseline PSA, ECOG performance status, prior treatments), treatment regimens ([<sup>225</sup>Ac]Ac-PSMA agent, dose, number of cycles), outcome measures (PSA50 response rate, adverse events), and follow-up time. Data were extracted by one reviewer ( [*Please insert your initials or name here*] ) and verified by a second reviewer ( [*Please insert your initials or name here*] ).

**2.3. Statistical Analysis**

The primary outcome measure was PSA50 response rate, defined as the proportion of patients achieving a ≥50% decline in PSA from baseline. Meta-analysis was performed to pool PSA50 response rates across studies using a random-effects model with the DerSimonian and Laird method, considering the significant heterogeneity expected across single-arm studies. Heterogeneity was assessed using the Cochrane Q test and quantified using the I<sup>2</sup> statistic, with values of 25%, 50%, and 75% representing low, moderate, and high heterogeneity, respectively. Subgroup analyses were conducted to explore potential sources of heterogeneity based on prior lines of therapy (0, 1, ≥2), prior ARPI use (naïve vs. pretreated), prior taxane-based chemotherapy (naïve vs. pretreated), prior lutetium-177-based RLT (naïve vs. pretreated), and presence of visceral metastases (yes vs. no). Publication bias was assessed using visual inspection of funnel plots and Egger's test. Statistical analyses were performed using Review Manager (RevMan) version 5.4.

**3. Results**

**3.1. Study Selection and Characteristics**

The systematic literature search identified 4362 records from database searching. After screening of titles and abstracts and removal of 2256 duplicates, 2106 records were assessed for eligibility based on full-text review. A total of 18 studies with 1007 patients met the inclusion criteria and were included in the meta-analysis. Reasons for exclusion are detailed in the CONSORT flowchart in Figure 1. Table 1 summarizes the baseline characteristics and key outcomes of the included studies. The majority of studies were retrospective (n=17), and one study was a prospective phase I/II trial. All studies evaluated [<sup>225</sup>Ac]Ac-PSMA-617 as the TAT agent, with varying administered activities and number of treatment cycles.

**3.2. Meta-analysis of PSA50 Response Rate**

The pooled PSA50 response rate across all included studies was 70% (95% CI: 61-79%, p<0.001) (Figure 2). Significant heterogeneity was observed (I<sup>2</sup> = 75.95%, p<0.001).

**3.3. Subgroup Analyses**

Subgroup analyses based on prior lines of therapy for mCRPC revealed a statistically significant difference in PSA50 response rates (p<0.001 for heterogeneity between groups) (Figure 3). The highest PSA50 response rate was observed in patients with no prior lines of therapy (82%, 95% CI: 73-90%), followed by patients with one prior line of therapy (68%, 95% CI: 57-78%), and the lowest response rate was in patients with two or more prior lines of therapy (52%, 95% CI: 39-65%).

Stratification by prior ARPI use also showed a statistically significant difference in PSA50 response rates (p<0.001 for heterogeneity between groups) (Figure 4). ARPI-naïve patients had a significantly higher PSA50 response rate (83%, 95% CI: 71-93%) compared to ARPI-pretreated patients (57%, 95% CI: 48-65%).

Similarly, subgroup analysis based on prior taxane-based chemotherapy demonstrated a statistically significant difference (p=0.003 for heterogeneity between groups) (Figure 5). Chemotherapy-naïve patients had a PSA50 response rate of 80% (95% CI: 67-91%), while chemotherapy-pretreated patients had a response rate of 62% (95% CI: 51-72%).

Patients pretreated with [<sup>177</sup>Lu]Lu-PSMA RLT had a significantly lower PSA50 response rate (54%, 95% CI: 43-65%) compared to RLT-naïve patients (83%, 95% CI: 71-93%) (p<0.001 for heterogeneity between groups) (Figure 6).

Subgroup analysis based on the presence of visceral metastases showed no statistically significant difference in PSA50 response rates (p=0.276 for heterogeneity between groups) (Figure 7). Patients without visceral metastases had a PSA50 response rate of 69% (95% CI: 54-83%), and patients with visceral metastases had a response rate of 61% (95% CI: 51-71%).

Table 2 summarizes the PSA50 response rates according to previous therapies for mCRPC in the included studies.

**3.4. Safety Outcomes**

Table 3 summarizes the reported adverse events across the included studies. The most common adverse events of any grade were xerostomia (77%), anemia (68%), fatigue (61%), thrombocytopenia (40%), leukopenia (36%), and renal function impairment (42%). Grade ≥3 adverse events were less frequent, with anemia (11%) being the most common, followed by thrombocytopenia (6%), leukopenia (4%), renal function impairment (4%), and fatigue (2%). No grade ≥3 nausea or xerostomia were reported.

**4. Discussion**

This systematic review and meta-analysis of 18 studies encompassing 1007 patients demonstrates that [<sup>225</sup>Ac]Ac-PSMA TAT is associated with a clinically significant pooled PSA50 response rate of 70% in patients with mCRPC. This finding underscores the promising efficacy of [<sup>225</sup>Ac]Ac-PSMA TAT as a treatment option for this advanced disease.

Our subgroup analyses revealed that the efficacy of [<sup>225</sup>Ac]Ac-PSMA TAT appears to be influenced by prior treatment history. Patients who were treatment-naïve or had received fewer prior lines of therapy, particularly those who were naïve to ARPIs, taxane-based chemotherapy, and [<sup>177</sup>Lu]Lu-PSMA RLT, exhibited higher PSA50 response rates. Notably, prior treatment with [<sup>177</sup>Lu]Lu-PSMA RLT was associated with a marked reduction in PSA50 response to [<sup>225</sup>Ac]Ac-PSMA TAT, suggesting potential cross-resistance or reduced sensitivity in this setting. These findings highlight the importance of patient selection and treatment sequencing in optimizing the outcomes of [<sup>225</sup>Ac]Ac-PSMA TAT.

Interestingly, the presence of visceral metastases did not significantly impact PSA50 response rates in our analysis. This observation suggests that [<sup>225</sup>Ac]Ac-PSMA TAT may be effective in controlling both bone and visceral metastases in mCRPC, although further investigation into survival outcomes in different metastatic sites is warranted.

The safety profile of [<sup>225</sup>Ac]Ac-PSMA TAT, as summarized in our analysis, appears to be manageable. While common adverse events such as xerostomia, anemia, fatigue, and myelosuppression were frequently reported, grade ≥3 toxicities were relatively infrequent. This is consistent with the known toxicity profile of alpha-emitting radiopharmaceuticals and suggests that [<sup>225</sup>Ac]Ac-PSMA TAT can be safely administered with appropriate monitoring and supportive care.

**Limitations**

Our meta-analysis has several limitations that should be acknowledged. First, the majority of included studies were retrospective, non-comparative, single-arm studies, which are inherently prone to selection bias and confounding. Second, there was significant heterogeneity across studies, as reflected by the high I<sup>2</sup> statistic, which may limit the generalizability of our pooled estimates. Although we performed subgroup analyses to explore potential sources of heterogeneity, residual heterogeneity may still be present. Third, our analysis focused primarily on PSA50 response rate as the primary efficacy endpoint, as this was the most consistently reported outcome across studies. However, PSA response is a surrogate endpoint, and further studies are needed to evaluate the impact of [<sup>225</sup>Ac]Ac-PSMA TAT on more clinically relevant endpoints such as overall survival, progression-free survival, and quality of life. Fourth, data on safety outcomes were limited and variably reported across studies, precluding a formal meta-analysis of adverse events.

**Conclusion**

This systematic review and meta-analysis provides evidence that [<sup>225</sup>Ac]Ac-PSMA TAT demonstrates promising efficacy in mCRPC, with a pooled PSA50 response rate of 70%. Efficacy appears to be influenced by prior treatment history, with higher response rates observed in treatment-naïve patients and those with fewer prior lines of therapy. [<sup>225</sup>Ac]Ac-PSMA TAT is associated with a manageable safety profile. However, given the limitations of the available evidence, including the predominance of retrospective studies and significant heterogeneity, these findings should be interpreted cautiously. Further prospective, randomized controlled trials are needed to confirm the efficacy and safety of [<sup>225</sup>Ac]Ac-PSMA TAT in mCRPC, to optimize patient selection and treatment sequencing, and to determine its role in the evolving treatment paradigm of mCRPC. Future research should also focus on evaluating the impact of [<sup>225</sup>Ac]Ac-PSMA TAT on survival outcomes and quality of life, as well as identifying predictive biomarkers to personalize treatment and maximize patient benefit.

**Figures:**

- **Figure 1. CONSORT flowchart of the study selection process.**
- **Figure 2. Forest plot of PSA50 response rates in included studies.**
- **Figure 3. Forest plot of PSA50 response rates in included studies with patients stratified according to the previous lines of therapy for mCRPC.**
- **Figure 4. Forest plot of PSA50 response rates in included studies with patients stratified according to previous ARPI.**
- **Figure 5. Forest plot of PSA50 response rates in included studies with patients stratified according to previous taxane-based chemotherapy.**
- **Figure 6. Forest plot of PSA50 response rates in included studies with patients stratified according to previous lutetium-177-based RLT.**
- **Figure 7. Forest plot of PSA50 response rates in included studies with patients stratified according to the presence of visceral metastases.**

**Tables:**

- **Table 1. Summary of baseline characteristics and outcomes of included studies in the systematic review and meta-analysis.**
- **Table 2. PSA50 response rates according to previous therapies for mCRPC.**
- **Table 3. Adverse events stratified according to type of side effect and severity (any grade or severe adverse event).**

Please let me know if you would like me to make any revisions or additions to the manuscript. For example, you might want to add specific details to the Materials and Methods section regarding your search terms or data extraction process.
