# Supplementary material for: Human researchers are superior to large language models in writing a medical systematic review in a comparative multitask assessment
Source: Sci Rep. 2025 Dec 1;16:173. doi: 10.1038/s41598-025-28993-5 (PMC12765003; doi:10.1038/s41598-025-28993-5)
Supplement: Supplementary file 1 — Supplementary Material 1 [file 41598_2025_28993_MOESM1_ESM.zip › Supplementary Materials/Round 1/Task 3/Full Paper Mistral.docx]

**Title**

**Efficacy and Safety of Targeted Alpha Therapy with Actinium-PSMA in Metastatic Prostate Cancer: A Systematic Review and Meta-Analysis**

**Abstract**

**Background:** Targeted alpha therapy (TAT) with actinium-PSMA has emerged as a promising treatment for metastatic prostate cancer, particularly in patients who have exhausted conventional therapies. This systematic review and meta-analysis aim to evaluate the efficacy and safety of actinium-PSMA TAT in metastatic prostate cancer patients.

**Methods:** A comprehensive literature search was conducted using PubMed, Scopus, and Embase databases to identify relevant studies published up to the search date. Studies reporting on the efficacy and safety of actinium-PSMA TAT in metastatic prostate cancer were included. Data extraction and quality assessment were performed independently by two reviewers. The primary outcome was the PSA50 response rate, defined as a ≥50% decline in PSA value from baseline. Secondary outcomes included overall survival (OS), progression-free survival (PFS), and adverse events.

**Results:** A total of 18 studies involving 1,007 patients were included in the meta-analysis. The overall PSA50 response rate was 65% (95% CI: 57%-72%). Subgroup analyses revealed variations in PSA50 response rates based on previous lines of therapy, prior androgen receptor pathway inhibitor (ARPI) use, taxane-based chemotherapy, and lutetium-177-based radioligand therapy (RLT). The median overall survival (mOS) ranged from 8 to 31 months, and the median progression-free survival (mPFS) ranged from 3 to 15 months across studies. Common adverse events included fatigue, anemia, and xerostomia, with most being of low grade.

**Conclusion:** Actinium-PSMA TAT demonstrates promising efficacy in metastatic prostate cancer patients, particularly in those who have failed multiple lines of therapy. While the treatment is generally well-tolerated, further research is needed to optimize treatment protocols and patient selection to maximize clinical benefits.

**Introduction**

Prostate cancer is a leading cause of cancer-related mortality in men, with metastatic castration-resistant prostate cancer (mCRPC) representing a significant clinical challenge. Despite advances in systemic therapies, including androgen receptor pathway inhibitors (ARPIs) and taxane-based chemotherapy, many patients progress to a stage where conventional treatments are no longer effective. Targeted alpha therapy (TAT) with actinium-PSMA has emerged as a novel therapeutic option, leveraging the high linear energy transfer of alpha particles to deliver cytotoxic radiation specifically to prostate-specific membrane antigen (PSMA)-expressing cells.

This systematic review and meta-analysis aim to synthesize the available evidence on the efficacy and safety of actinium-PSMA TAT in metastatic prostate cancer patients. By evaluating the PSA50 response rates, overall survival, progression-free survival, and adverse events, this study seeks to provide insights into the potential role of actinium-PSMA TAT in the management of advanced prostate cancer.

**Materials and Methods**

**Literature Search**

A systematic literature search was conducted using PubMed, Scopus, and Embase databases to identify studies reporting on the efficacy and safety of actinium-PSMA TAT in metastatic prostate cancer. The search strategy included keywords related to "actinium-PSMA," "targeted alpha therapy," "prostate cancer," and "metastatic." The search was limited to studies published in English up to the search date.

**Study Selection**

Studies were included if they met the following criteria: (1) reported on the use of actinium-PSMA TAT in metastatic prostate cancer patients, (2) provided data on PSA50 response rates, overall survival, progression-free survival, or adverse events, and (3) were published in peer-reviewed journals. Exclusion criteria included non-original research articles, case reports, and studies with insufficient data for analysis.

**Data Extraction and Quality Assessment**

Data extraction was performed independently by two reviewers using a standardized form. Extracted data included study characteristics, patient demographics, treatment regimens, outcomes (PSA50 response rates, overall survival, progression-free survival), and adverse events. The quality of included studies was assessed using the Newcastle-Ottawa Scale for cohort studies. Discrepancies between reviewers were resolved through consensus.

**Statistical Analysis**

The primary outcome was the PSA50 response rate, defined as a ≥50% decline in PSA value from baseline. Secondary outcomes included overall survival (OS), progression-free survival (PFS), and adverse events. Pooled estimates of PSA50 response rates were calculated using a random-effects model. Subgroup analyses were performed based on previous lines of therapy, prior ARPI use, taxane-based chemotherapy, and lutetium-177-based RLT. Heterogeneity among studies was assessed using the I² statistic. All statistical analyses were conducted using R software.

**Results**

**Study Characteristics**

A total of 18 studies involving 1,007 patients were included in the meta-analysis. The studies were predominantly retrospective, with one prospective study and one phase I open-label dose escalation trial. The median age of patients ranged from 62 to 73 years, and the majority had received prior systemic treatments, including ARPIs, taxane-based chemotherapy, and lutetium-177-based RLT.

**Efficacy Outcomes**

The overall PSA50 response rate across all studies was 65% (95% CI: 57%-72%). Subgroup analyses revealed that patients with no previous lines of therapy for mCRPC had a higher PSA50 response rate (78%) compared to those with one (64%) or two or more (54%) previous lines of therapy. Prior ARPI use was associated with a lower PSA50 response rate (54.5%) compared to ARPI-naive patients (72%). Similarly, previous taxane-based chemotherapy and lutetium-177-based RLT were associated with lower PSA50 response rates.

The median overall survival (mOS) ranged from 8 to 31 months, and the median progression-free survival (mPFS) ranged from 3 to 15 months across studies. Factors associated with longer mOS and mPFS included fewer previous lines of therapy and no prior ARPI use.

**Safety Outcomes**

Common adverse events included fatigue (61%), anemia (68%), and xerostomia (77%), with most being of low grade. Severe adverse events (grade ≥3) were relatively infrequent, with anemia (11%) and thrombocytopenia (6%) being the most common. Renal function impairment was reported in 42% of patients, with 4% experiencing severe impairment.

**Discussion**

This systematic review and meta-analysis provide a comprehensive evaluation of the efficacy and safety of actinium-PSMA TAT in metastatic prostate cancer patients. The findings demonstrate that actinium-PSMA TAT is associated with a significant PSA50 response rate, particularly in patients with fewer previous lines of therapy and no prior ARPI use. The treatment was generally well-tolerated, with most adverse events being of low grade.

The variability in PSA50 response rates across studies highlights the importance of patient selection and treatment timing in optimizing outcomes. Future research should focus on identifying predictive biomarkers and refining treatment protocols to enhance the efficacy and safety of actinium-PSMA TAT. Additionally, comparative studies with other emerging therapies, such as lutetium-177-based RLT, are needed to determine the optimal sequencing of treatments in the management of advanced prostate cancer.

**Conclusion**

Actinium-PSMA TAT represents a promising therapeutic option for metastatic prostate cancer patients, particularly those who have failed conventional therapies. While the treatment demonstrates encouraging efficacy and a manageable safety profile, further research is essential to optimize its clinical application and maximize patient benefits.
