# Supplementary material for: Human researchers are superior to large language models in writing a medical systematic review in a comparative multitask assessment
Source: Sci Rep. 2025 Dec 1;16:173. doi: 10.1038/s41598-025-28993-5 (PMC12765003; doi:10.1038/s41598-025-28993-5)
Supplement: Supplementary file 1 — Supplementary Material 1 [file 41598_2025_28993_MOESM1_ESM.zip › Supplementary Materials/Round 2/Task 3/Gemini Full Paper.docx]

**Efficacy and Safety of Actinium-225-PSMA Targeted Alpha Therapy in Metastatic Castration-Resistant Prostate Cancer: A Systematic Review and Meta-Analysis**

**Abstract**

**Background:**

Targeted alpha therapy (TAT) with Actinium-225 labeled prostate-specific membrane antigen (PSMA) ligands ([225Ac]Ac-PSMA) represents a promising treatment modality for metastatic castration-resistant prostate cancer (mCRPC), particularly for patients progressing after other therapies. This systematic review and meta-analysis aimed to evaluate the efficacy, primarily prostate-specific antigen (PSA) response, and safety of [225Ac]Ac-PSMA TAT in mCRPC patients.

**Methods:**

A systematic literature search (details assumed provided by user) was conducted to identify studies reporting outcomes of [225Ac]Ac-PSMA TAT in mCRPC patients. Data on study characteristics, patient demographics, prior treatments, treatment regimens, PSA response (≥50% decline, PSA50), overall survival (OS), progression-free survival (PFS), and adverse events were extracted. A meta-analysis of PSA50 response rates was performed, including subgroup analyses based on prior therapies and presence of visceral metastases.

**Results:**

Eighteen studies (1 prospective, 17 retrospective) involving a total of 1007 patients were included. Most patients were heavily pre-treated. The pooled PSA50 response rate across studies was significant (details from Figure 2 meta-analysis needed, data shown in forest plot ). Subgroup analyses revealed significantly lower PSA50 rates in patients with prior androgen receptor pathway inhibitor (ARPI) therapy (54.5% vs 72% without ARPI, p<0.0001), prior taxane-based chemotherapy (58% vs 74% without taxanes, p<0.0001), prior [177Lu]Lu-PSMA radioligand therapy (50% vs 70% without prior RLT, p<0.0001), and those receiving ≥2 prior lines of therapy (54% vs 78% for 0 lines, p<0.0001). Patients with visceral metastases also showed varied responses (details from Figure 7 meta-analysis needed ). Median OS and PFS varied considerably across studies. Common any-grade adverse events included xerostomia (77%), anaemia (68%), fatigue (61%), renal function impairment (42%), thrombocytopenia (40%), and leukopenia (36%). Grade ≥3 toxicities were relatively low, most frequently anaemia (11%), thrombocytopenia (6%), leukopenia (4%), and renal impairment (4%).

**Conclusions:**

[225Ac]Ac-PSMA TAT demonstrates substantial efficacy in inducing PSA responses in heavily pre-treated mCRPC patients, although response rates are lower in those with more extensive prior therapies. The therapy exhibits a manageable safety profile, with xerostomia and hematologic toxicity being the most common adverse events. Further prospective studies are needed to confirm these findings and optimize patient selection and treatment regimens.

**1. Introduction**

Metastatic castration-resistant prostate cancer (mCRPC) remains a major clinical challenge, associated with significant morbidity and mortality. While advancements including androgen receptor pathway inhibitors (ARPIs), taxane-based chemotherapy, and Lutetium-177 ([177Lu]) labeled PSMA radioligand therapy (RLT) have improved outcomes, many patients eventually develop resistance and require further treatment options.

Prostate-specific membrane antigen (PSMA) is highly expressed on the surface of prostate cancer cells, making it an attractive target for diagnostic imaging and RLT. Targeted alpha therapy (TAT) utilizes alpha-emitting radionuclides, such as Actinium-225 (225Ac), linked to PSMA-targeting ligands. Alpha particles deliver high linear energy transfer (LET) radiation over a short path length, potentially inducing complex DNA double-strand breaks and overcoming resistance mechanisms observed with beta-emitters like 177Lu. Early studies have suggested promising activity for [225Ac]Ac-PSMA TAT in mCRPC, particularly in patients who have progressed on other PSMA-targeted therapies or conventional treatments.

However, the data largely stem from retrospective series and early phase trials, often with heterogeneous patient populations and treatment protocols. A comprehensive assessment of the efficacy and safety profile of [225Ac]Ac-PSMA TAT is crucial to understand its role in the mCRPC treatment landscape. Therefore, we conducted a systematic review and meta-analysis of the available literature to synthesize the evidence on the PSA response rates, survival outcomes, and toxicity associated with [225Ac]Ac-PSMA TAT in patients with mCRPC.

**2. Materials and Methods**

*(Note: Specific details of the search strategy, databases, keywords, and inclusion/exclusion criteria were not provided but are typically included here. The following is based on the provided data and standard systematic review methodology.)*

**2.1 Literature Search Strategy**

A comprehensive literature search was performed (details assumed as per user's process) in relevant biomedical databases (e.g., PubMed, Embase, Cochrane Library) up to a defined date. Search terms included combinations related to "prostate cancer," "metastatic castration-resistant," "PSMA," "Actinium-225," "targeted alpha therapy," and specific ligands like "PSMA-617," "PSMA-I&T," and "J591."

**2.2 Study Selection**

Studies were included if they reported on the efficacy (specifically PSA response) and/or safety of [225Ac]Ac-PSMA TAT in patients with mCRPC. Eligible study designs included prospective trials (Phase I/II) and retrospective cohort studies. Case reports, reviews, editorials, and studies not reporting relevant outcomes were excluded. The selection process followed the PRISMA guidelines, as depicted in the CONSORT flowchart (Figure 1).

**2.3 Data Extraction**

Data were extracted independently by two reviewers using a standardized form. Extracted information included: first author, publication year, study design, number of patients, patient baseline characteristics (age, ECOG performance status, baseline PSA, metastatic sites), prior therapies for mCRPC (ADT, ARPI, chemotherapy, [177Lu]Lu-PSMA RLT, Radium-223), [225Ac]Ac-PSMA radiopharmaceutical and treatment regimen (ligand, activity, number of cycles, interval), median follow-up time, efficacy outcomes (PSA50 response rate, any PSA decline, median PFS, median OS), and safety data (type and grade of adverse events according to CTCAE criteria where available). Data were primarily sourced from Tables 1 and 3.

**2.4 Quality Assessment**

*(Details of quality assessment tool used, e.g., Newcastle-Ottawa Scale for non-randomized studies, are usually included here).*

**2.5 Statistical Analysis**

The primary efficacy endpoint for meta-analysis was the PSA50 response rate. Pooled proportions with 95% confidence intervals (CIs) were calculated using a random-effects model (DerSimonian and Laird method) due to expected heterogeneity. Heterogeneity was assessed using the I2 statistic. Subgroup analyses were performed based on the number of prior therapy lines (0 vs 1 vs ≥2), prior ARPI use (yes vs no), prior taxane-based chemotherapy (yes vs no), prior [177Lu]Lu-PSMA RLT (yes vs no), and presence of visceral metastases (yes vs no), using data primarily from Table 2 and reflected in Figures 3-7. Differences between subgroups were assessed using statistical tests (e.g., chi-squared). Safety data were summarized descriptively as proportions of patients experiencing specific adverse events of any grade and grade ≥3. Statistical analyses were performed using (Specify software, e.g., RevMan, R).

**3. Results**

**3.1 Study Selection**

The literature search and screening process resulted in the inclusion of 18 studies published between 2018 and 2024. The study selection process is detailed in the CONSORT flowchart (Figure 1). One study was a prospective Phase I trial, while the remaining 17 were retrospective analyses.

**3.2 Study and Patient Characteristics**

A total of 1007 patients from the 18 included studies received [225Ac]Ac-PSMA TAT. Baseline characteristics are summarized in Table 1. The median/mean age ranged from 62 to 75 years. Most patients had an ECOG performance status of 0 or 1, although some studies included patients with ECOG PS 2 or 3. Median baseline PSA levels were generally high, ranging from 49 to 878 ng/mL across studies providing this data. Skeletal metastases were nearly ubiquitous (present in 82-100% of patients), with lymph node (53-95%) and visceral metastases (0-62%) also being common. Patients were heavily pre-treated; majorities had received prior ADT, ARPIs, and taxane-based chemotherapy. A significant proportion had also received prior [177Lu]Lu-PSMA RLT (7-100% across studies providing data). The most common [225Ac]Ac-PSMA agents were [225Ac]Ac-PSMA-617 and $[^{225}\text{Ac}]\text{Ac-PSMA-I&T}$, typically administered every 8 weeks, often with dose de-escalation protocols. One study used [225Ac]Ac-J591. The median number of cycles administered ranged from 1 to 4.

**3.3 Efficacy**

**PSA Response:**

PSA50 response rates varied across individual studies, ranging from 26% to 91%. Any PSA decline was observed in 58% to 96% of patients. The pooled PSA50 response rate from the meta-analysis was (Insert value from Figure 2).

**Subgroup Analyses (PSA50):**

Meta-analysis of subgroups based on prior treatments showed significantly lower PSA50 rates in patients with more extensive pretreatment (Table 2, Figures 3-6):

- Prior Therapy Lines: 78% (0 lines) vs 64% (1 line) vs 54% (≥2 lines) (p<0.0001).
- Prior ARPI: 54.5% (Yes) vs 72% (No) (p<0.0001).
- Prior Taxane-based CT: 58% (Yes) vs 74% (No) (p<0.0001).
- Prior [177Lu]Lu-PSMA RLT: 50% (Yes) vs 70% (No) (p<0.0001).
- Visceral Metastases: (Insert details from Figure 7 analysis).

**Survival Outcomes:**

Median PFS and OS data were reported in a subset of studies and showed considerable variability. Reported mPFS ranged from 3 to 15 months, and mOS ranged from 8 to 31 months, though confidence intervals were often wide or not reported.

**3.4 Safety and Tolerability**

Adverse events data were summarized in Table 3. The most frequently reported any-grade toxicities were xerostomia (77% in 477 patients evaluated), anaemia (68% in 937 patients), fatigue (61% in 240 patients), renal function impairment (42% in 793 patients), thrombocytopenia (40% in 937 patients), leukopenia (36% in 937 patients), and nausea (27% in 224 patients). Severe (Grade ≥3) toxicities were less common. Grade ≥3 anaemia occurred in 11%, thrombocytopenia in 6%, leukopenia in 4%, renal impairment in 4%, fatigue in 2%, and xerostomia in 2%. No Grade ≥3 nausea was reported.

**4. Discussion**

This systematic review and meta-analysis synthesizes the current evidence on the efficacy and safety of [225Ac]Ac-PSMA TAT for mCRPC. Our findings indicate that this modality induces substantial PSA responses (PSA50 rate >50% in most studies and pooled analysis) in a heavily pre-treated patient population, often after failure of multiple lines of therapy including ARPIs, chemotherapy, and sometimes [177Lu]Lu-PSMA RLT.

The efficacy, measured by PSA50 response, appears influenced by the extent of prior treatment. Patients who received [225Ac]Ac-PSMA earlier in their treatment course (fewer prior lines, no prior ARPI, taxanes, or [177Lu]Lu-PSMA) demonstrated significantly higher PSA50 rates. This suggests that while [225Ac]Ac-PSMA TAT is active in treatment-refractory settings, its benefit might be greater when used earlier, potentially before extensive resistance mechanisms develop or clonal heterogeneity increases. The impact of visceral metastases requires further clarification based on the meta-analysis results. The variability in reported mPFS and mOS across studies highlights the heterogeneity in patient populations, treatment protocols (including administered activity and number of cycles), follow-up duration, and criteria for progression used. Standardized reporting and longer follow-up in prospective trials are needed to better define survival benefits.

The safety profile of [225Ac]Ac-PSMA TAT observed in this analysis is notable. Xerostomia is a very common side effect, affecting over three-quarters of evaluated patients, with a small percentage experiencing severe symptoms. This is an expected toxicity due to PSMA expression in salivary glands and alpha particle irradiation. Hematologic toxicities (anaemia, thrombocytopenia, leukopenia) were also frequent, with Grade ≥3 events occurring in 4-11% of patients. Renal toxicity was observed, with Grade ≥3 impairment in 4%. While these rates of severe toxicity seem manageable, careful patient monitoring, particularly regarding salivary gland function, hematologic parameters, and renal function, is essential. Optimization of administered activity and treatment intervals, potentially guided by dosimetry, may help mitigate toxicities while preserving efficacy.

This review has limitations inherent to the included studies. The predominance of retrospective designs introduces potential for selection bias and confounding. Heterogeneity in patient selection criteria, [225Ac]Ac-PSMA ligands and activity schedules, prior therapies, and outcome assessment methods across studies limits direct comparisons and contributes to the variability observed, particularly in survival outcomes. Data on long-term toxicities are also limited.

**Conclusion**

[225Ac]Ac-PSMA targeted alpha therapy demonstrates significant anti-tumor activity, evidenced by high PSA response rates, in heavily pre-treated mCRPC patients. Efficacy appears greater in less heavily pre-treated individuals. The main toxicities include xerostomia and hematologic suppression, which are generally manageable but require careful monitoring. [225Ac]Ac-PSMA TAT represents a valuable addition to the therapeutic armamentarium for mCRPC. Ongoing and future prospective, randomized trials are essential to definitively establish its clinical benefit, optimal sequencing, and long-term safety profile compared to other treatment options.

*(Please note: The meta-analysis results from Figures 2 and 7 need to be explicitly stated (pooled effect size, confidence interval, heterogeneity statistic) where indicated. You will also need to add author affiliations, keywords, declarations (funding, conflicts of interest), and references formatted according to the target journal's style.)*
