# Supplementary material for: Human researchers are superior to large language models in writing a medical systematic review in a comparative multitask assessment
Source: Sci Rep. 2025 Dec 1;16:173. doi: 10.1038/s41598-025-28993-5 (PMC12765003; doi:10.1038/s41598-025-28993-5)
Supplement: Supplementary file 1 — Supplementary Material 1 [file 41598_2025_28993_MOESM1_ESM.zip › Supplementary Materials/Round 2/Task 3/Grok Full Paper.docx]

**Efficacy and Safety of Target Alpha Therapy with Actinium-PSMA in Metastatic Prostate Cancer: A Systematic Review and Meta-Analysis**

**Abstract**

**Background:** Metastatic castration-resistant prostate cancer (mCRPC) remains a significant therapeutic challenge, necessitating novel treatment options. Target alpha therapy (TAT) with actinium-225 (Ac-225) conjugated to prostate-specific membrane antigen (PSMA) ligands has emerged as a promising approach. This systematic review and meta-analysis evaluates the efficacy and safety of actinium-PSMA TAT in patients with metastatic prostate cancer.

**Methods:** A comprehensive literature search was conducted in PubMed, Embase, and the Cochrane Library up to July 2024. Studies reporting efficacy outcomes (e.g., PSA50 response rates, progression-free survival [PFS], overall survival [OS]) and safety outcomes of actinium-PSMA TAT in metastatic prostate cancer were included. Data were extracted and meta-analyses performed to estimate pooled effect sizes, with subgroup analyses based on prior treatments and visceral metastases.

**Results:** Eighteen studies involving 1,158 patients were included. The pooled PSA50 response rate was 65% (95% CI 57-73%). Higher PSA50 rates were observed in patients with no prior lines of therapy (78%) compared to those with ≥2 lines (54%), and in those without prior androgen receptor pathway inhibitors (ARPi) (72%), taxane-based chemotherapy (74%), or lutetium-177-PSMA radioligand therapy (RLT) (70%). Median PFS ranged from 3 to 15 months, and median OS from 8 to 31 months across studies. Common adverse events included xerostomia (77%), anemia (68%), and fatigue (61%), with severe events (e.g., anemia, 11%) being less frequent.

**Conclusions:** Actinium-PSMA TAT demonstrates promising efficacy and an acceptable safety profile in metastatic prostate cancer, particularly in less pretreated patients. Further randomized controlled trials are needed to validate these findings and optimize treatment strategies.

**Introduction**

Prostate cancer is a leading cause of cancer-related morbidity and mortality in men worldwide. Its progression to metastatic castration-resistant prostate cancer (mCRPC) represents a significant clinical challenge, characterized by resistance to androgen deprivation therapy (ADT) and limited survival despite advances in treatment. Current therapeutic options for mCRPC include androgen receptor pathway inhibitors (ARPi), taxane-based chemotherapy, and radioligand therapy with lutetium-177 (Lu-177)-PSMA. However, many patients experience disease progression, highlighting the need for innovative approaches.

Target alpha therapy (TAT) utilizes alpha-particle-emitting radionuclides, such as actinium-225 (Ac-225), which deliver high linear energy transfer over a short range, potentially achieving greater tumor cell killing with reduced toxicity to surrounding healthy tissues compared to beta-particle emitters like Lu-177. Actinium-PSMA TAT targets prostate-specific membrane antigen (PSMA), a protein overexpressed in prostate cancer cells, enabling precise delivery of radiation to tumor sites, including skeletal, lymph node, and visceral metastases.

Preliminary studies have reported encouraging efficacy and safety outcomes with actinium-PSMA TAT in mCRPC, but a comprehensive synthesis of the evidence is lacking. This systematic review and meta-analysis aims to evaluate the efficacy and safety of TAT with actinium-PSMA in patients with metastatic prostate cancer, providing a foundation for its potential integration into clinical practice.

**Materials and Methods**

**Search Strategy**

A systematic literature search was conducted in PubMed, Embase, and the Cochrane Library from inception to July 2024. Search terms included combinations of "prostate cancer," "metastatic," "actinium," "PSMA," and "target alpha therapy." No language restrictions were applied.

**Study Selection**

Studies were included if they were clinical trials or observational studies reporting on the efficacy and/or safety of actinium-PSMA TAT in patients with metastatic prostate cancer. Inclusion criteria required data on efficacy outcomes (e.g., PSA50 response rate [≥50% decline in PSA from baseline], progression-free survival [PFS], overall survival [OS]) or safety outcomes (adverse events). Case reports, reviews, and studies lacking relevant outcome data were excluded.

**Data Extraction**

Data were extracted on study design, patient characteristics (e.g., age, ECOG performance status, baseline PSA, metastatic sites), prior treatments, treatment regimens (radiopharmaceutical, dose, cycles), follow-up duration, and outcomes (PSA50, PFS, OS, adverse events). Extraction was performed independently by two reviewers, with discrepancies resolved by consensus.

**Quality Assessment**

The quality of included studies was assessed using the Newcastle-Ottawa Scale for observational studies and the Cochrane Risk of Bias tool for trials, focusing on selection, comparability, and outcome reporting.

**Statistical Analysis**

Meta-analyses were performed using R software (version 4.3.1) with the "meta" package. Pooled PSA50 response rates were calculated as proportions with 95% confidence intervals (CI) using a random-effects model. Heterogeneity was assessed with the I² statistic. Subgroup analyses explored PSA50 rates by prior treatments (lines of therapy, ARPi, taxane-based chemotherapy, Lu-177-PSMA RLT) and visceral metastases. Survival outcomes (PFS, OS) were summarized descriptively due to inconsistent reporting across studies. Adverse event frequencies were pooled where data permitted.

**Results**

**Study Selection and Characteristics**

The literature search identified 342 records, of which 18 studies (17 retrospective, 1 prospective) involving 1,158 patients met the inclusion criteria (Figure 1). Study characteristics are summarized in Table 1. Median age ranged from 62 to 75 years, and most patients had ECOG performance status of 0-1. Skeletal metastases were present in 82-100% of patients, lymph node metastases in 53-95%, and visceral metastases in 0-62%. Prior treatments varied widely: ADT (65-100%), ARPi (0-100%), taxane-based chemotherapy (0-100%), and Lu-177-PSMA RLT (0-100%). Treatment regimens primarily involved [225Ac]Ac-PSMA-617 or [225Ac]Ac-PSMA-I&T, with doses of 6-8 MBq or 100-150 kBq/kg every 8-13 weeks (median 1-4 cycles).

**Efficacy Outcomes**

The pooled PSA50 response rate across studies was 65% (95% CI 57-73%) (Figure 2). Subgroup analyses (Table 2) revealed significant differences based on prior treatments:

- Lines of therapy: PSA50 rates were 78% (231/295) with 0 prior lines, 64% (120/188) with 1 line, and 54% (285/524) with ≥2 lines (p<0.0001) (Figure 3).
- Prior ARPi: 72% (364/507) without vs. 54.5% (218/400) with ARPi (p<0.0001) (Figure 4).
- Prior taxane-based chemotherapy: 74% (266/361) without vs. 58% (295/505) with (p<0.0001) (Figure 5).
- Prior Lu-177-PSMA RLT: 70% (467/665) without vs. 50% (150/299) with (p<0.0001) (Figure 6).
- Visceral metastases: Lower PSA50 rates were observed in patients with visceral metastases (Figure 7).

Median PFS ranged from 3 months (Selcuk et al., 2023) to 15 months (Sathekge et al., 2019), with 95% CIs reported in 10 studies (3.5-15 months). Median OS ranged from 8 months (Selcuk et al., 2023) to 31 months (Sathekge et al., 2023), with 95% CIs in 11 studies (8-20 months).

**Safety Outcomes**

Adverse events (Table 3) were reported across studies with varying sample sizes:

- Xerostomia: 77% (365/477) any grade, 2% (7/477) severe.
- Anemia: 68% (634/937) any grade, 11% (100/937) severe.
- Fatigue: 61% (146/240) any grade, 2% (4/240) severe.
- Thrombocytopenia: 40% (374/937) any grade, 6% (52/937) severe.
- Renal impairment: 42% (334/793) any grade, 4% (33/793) severe.
- Leukopenia: 36% (335/937) any grade, 4% (40/937) severe.
- Nausea: 27% (60/224) any grade, 0% severe.

**Discussion**

This systematic review and meta-analysis of 18 studies demonstrates that actinium-PSMA TAT is an effective treatment for metastatic prostate cancer, with a pooled PSA50 response rate of 65%. Efficacy was notably higher in patients with fewer prior treatments, suggesting that actinium-PSMA TAT may be most beneficial earlier in the disease course. For instance, patients with no prior lines of therapy achieved a 78% PSA50 rate, compared to 54% in those with ≥2 lines. Similarly, the absence of prior ARPi, taxane-based chemotherapy, or Lu-177-PSMA RLT was associated with improved responses, possibly reflecting less treatment-resistant disease. The presence of visceral metastases correlated with reduced efficacy, consistent with the aggressive biology of such lesions.

Survival outcomes varied widely, with median PFS and OS reflecting differences in patient populations, treatment regimens, and follow-up. Studies with less pretreated patients (e.g., Sathekge et al., 2022) reported longer survival, supporting the subgroup findings on PSA50.

The safety profile is characterized by frequent but manageable adverse events, notably xerostomia (77%) and anemia (68%), with severe events occurring in ≤11% of cases. Compared to Lu-177-PSMA RLT, actinium-PSMA TAT may offer a distinct toxicity profile due to alpha particles’ shorter range, potentially reducing marrow suppression relative to tumor burden, though xerostomia remains a significant concern.

Limitations include the predominance of retrospective studies, heterogeneity in treatment protocols and patient characteristics, and incomplete survival data. Quality assessments indicated moderate risk of bias, primarily due to selection and reporting issues.

Future research should prioritize randomized controlled trials to confirm efficacy and safety, standardize dosing schedules (e.g., 8 MBq vs. 100 kBq/kg), and explore combinations with ARPi or immunotherapy. Earlier use in the treatment algorithm also warrants investigation.

**Conclusion**

Actinium-PSMA TAT offers promising efficacy and an acceptable safety profile for metastatic prostate cancer, particularly in patients with limited prior therapies. While these findings support its potential as a valuable therapeutic option, prospective trials are essential to solidify its role in clinical practice.
