# Supplementary material for: Human researchers are superior to large language models in writing a medical systematic review in a comparative multitask assessment
Source: Sci Rep. 2025 Dec 1;16:173. doi: 10.1038/s41598-025-28993-5 (PMC12765003; doi:10.1038/s41598-025-28993-5)
Supplement: Supplementary file 1 — Supplementary Material 1 [file 41598_2025_28993_MOESM1_ESM.zip › Supplementary Materials/Round 2/Task 3/ChatGPT Full Paper.docx]

**Abstract**

**Background:** Targeted Alpha Therapy (TAT) using Actinium-225-labelled prostate-specific membrane antigen (Ac-PSMA) has emerged as a promising treatment for metastatic castration-resistant prostate cancer (mCRPC). This systematic review and meta-analysis aimed to evaluate the efficacy and safety of Ac-PSMA therapy in patients with mCRPC.

**Methods:** Systematic literature searches were performed, selecting studies evaluating the efficacy (PSA reduction, progression-free survival (PFS), overall survival (OS)) and safety of Ac-PSMA in mCRPC. Meta-analyses were conducted using random-effects models, assessing the PSA response rates (PSA50) stratified by previous treatments and metastasis sites.

**Results:** Seventeen studies comprising 1,007 patients were included. The pooled PSA50 response rate was significant across studies, varying according to prior therapies: PSA50 response was highest in treatment-naive patients (78%) and lower after multiple previous treatments (54%) (p<0.0001). Prior use of androgen receptor pathway inhibitors (ARPi), taxane-based chemotherapy, or Lutetium-177-based therapy significantly influenced the PSA50 responses (p<0.0001 for all). Median OS ranged from 8 to 31 months, with median PFS ranging from 3 to 15 months. Adverse events included fatigue (61%), anaemia (68%), xerostomia (77%), and hematological toxicity, with severe events being relatively rare.

**Conclusion:** Ac-PSMA therapy demonstrates substantial efficacy in PSA reduction and prolonged survival outcomes in patients with mCRPC, particularly when used early in the treatment sequence. Despite the frequent occurrence of adverse effects, severe events are uncommon, underscoring the acceptable safety profile of this therapy.

**Introduction**

Metastatic castration-resistant prostate cancer (mCRPC) poses significant clinical management challenges due to its resistance to conventional therapies. Targeted Alpha Therapy (TAT), particularly utilizing Actinium-225-labelled prostate-specific membrane antigen (Ac-PSMA), has gained attention due to its potent therapeutic effects via targeted radiation delivery. This systematic review and meta-analysis assesses the current literature on the efficacy and safety of Ac-PSMA therapy in patients with mCRPC.

**Materials and Methods**

A comprehensive literature search was conducted across major databases (PubMed, EMBASE, Cochrane) to identify relevant studies evaluating the efficacy and safety of Ac-PSMA therapy. Studies were selected based on predefined eligibility criteria, and data extraction included patient demographics, previous treatments, treatment regimens, and clinical outcomes. Quality assessment was performed, and meta-analysis employed random-effects models, focusing on the proportion of patients achieving a ≥50% decline in PSA (PSA50), stratified by previous therapies and metastasis characteristics.

**Results**

**A total of 17 studies involving 1,007 patients were included. Patient characteristics indicated an advanced disease stage, with significant prior exposure to systemic** therapies. The pooled analysis showed robust PSA50 responses, highest in treatment-naive patients (78%) and decreasing with increasing numbers of previous therapies. The meta-analysis stratified by treatment history demonstrated significant differences (all p<0.0001), with notably higher PSA50 responses observed in patients without prior ARPi or taxane-based chemotherapy.

Median progression-free survival ranged widely from 3 to 15 months, and median overall survival varied significantly from 8 to 31 months. Adverse events were frequently observed, particularly fatigue (61%), anaemia (68%), leukopenia (36%), thrombocytopenia (40%), renal impairment (42%), and xerostomia (77%). Severe adverse events were uncommon, with grade ≥3 anaemia being most frequent (11%).

**Discussion**

The results of this review affirm the potent therapeutic efficacy of Ac-PSMA in patients with mCRPC, particularly when administered early in the therapeutic sequence. PSA response rates and survival outcomes clearly depend on prior treatment exposure, emphasizing the potential advantages of earlier introduction of Ac-PSMA therapy.

Despite a relatively high incidence of adverse events, the overall safety profile remains acceptable, with most toxicities being manageable. The predominance of mild to moderate adverse effects suggests a favorable therapeutic index, making Ac-PSMA a viable option in the therapeutic landscape of advanced prostate cancer.

Future research should focus on randomized controlled trials to confirm these findings and optimize patient selection and treatment sequencing to maximize therapeutic benefits while minimizing adverse effects.

**Conclusion**

Actinium-PSMA TAT offers significant therapeutic benefits with acceptable safety for patients with metastatic castration-resistant prostate cancer, particularly when used early in disease management. Prospective randomized studies are warranted to further define its role in clinical practice.
