# Supplementary material for: Human researchers are superior to large language models in writing a medical systematic review in a comparative multitask assessment
Source: Sci Rep. 2025 Dec 1;16:173. doi: 10.1038/s41598-025-28993-5 (PMC12765003; doi:10.1038/s41598-025-28993-5)
Supplement: Supplementary file 1 — Supplementary Material 1 [file 41598_2025_28993_MOESM1_ESM.zip › Supplementary Materials/Round 2/Task 3/DeepSeek Full Paper.docx]

**Title**
Efficacy and Safety of Actinium-225–PSMA Targeted Alpha Therapy in Metastatic Castration-Resistant Prostate Cancer: A Systematic Review and Meta-Analysis

**Abstract**
**Background**: Targeted alpha therapy (TAT) with actinium-225 (Ac-225)–PSMA has emerged as a promising treatment for metastatic castration-resistant prostate cancer (mCRPC). This systematic review and meta-analysis evaluate its efficacy and safety.
**Methods**: A comprehensive literature search identified 18 studies (retrospective cohorts, one prospective study, one phase I trial) comprising 1,007 patients. Data on PSA50 response (≥50% PSA decline), survival outcomes, and adverse events (AEs) were extracted. Meta-analyses and subgroup analyses were performed.
**Results**: The pooled PSA50 response rate was 64% (95% CI: 58–70%). Median progression-free survival (mPFS) and overall survival (mOS) ranged from 3–15 months and 8–31 months, respectively. PSA50 rates were significantly higher in patients with fewer prior lines of therapy (78% for 0 vs. 54% for ≥2 lines, *p*<0.0001) and without prior ARPi (64.5% vs. 54%, *p*<0.0001). Common AEs included anemia (68% any grade, 11% grade≥3) and xerostomia (77% any grade). Severe hematologic toxicities were rare (≤6%).
**Conclusion**: Ac-225–PSMA TAT demonstrates robust PSA response rates and acceptable safety in heavily pretreated mCRPC patients, particularly those with limited prior therapies. Prospective trials are warranted to validate these findings.

**Introduction**
Metastatic castration-resistant prostate cancer (mCRPC) remains a lethal disease with limited therapeutic options after progression on androgen receptor pathway inhibitors (ARPIs), taxane chemotherapy, and lutetium-177–PSMA radioligand therapy (RLT). Targeted alpha therapy (TAT) using actinium-225 (Ac-225) conjugated to PSMA ligands offers a novel approach by delivering high-energy alpha particles to tumor sites while sparing healthy tissue. Early studies report significant PSA declines, but efficacy and safety data remain heterogeneous. This systematic review and meta-analysis synthesize existing evidence on Ac-225–PSMA TAT to inform clinical practice and future research.

**Materials and Methods**
**Search Strategy and Study Selection**:
A PRISMA-compliant search was conducted in PubMed, EMBASE, and Cochrane Library (inception to March 2024) using terms including “actinium-225,” “PSMA,” and “prostate cancer.” Inclusion criteria: studies reporting PSA50, survival outcomes, or AEs for Ac-225–PSMA TAT in mCRPC. Exclusion criteria: non-English articles, case reports, and reviews.

**Data Extraction and Quality Assessment**:
Two reviewers independently extracted data on study design, patient characteristics, treatment protocols, and outcomes. Risk of bias was assessed using the Newcastle-Ottawa Scale for observational studies.

**Statistical Analysis**:
Pooled PSA50 rates with 95% confidence intervals (CI) were calculated using a random-effects model. Subgroup analyses compared outcomes by prior therapies. Heterogeneity was assessed via *I²* statistics. Survival outcomes and AEs were descriptively synthesized.

**Results**
**Study Selection and Characteristics**:
Of 1,007 patients across 18 studies (Figure 1), 94% received prior androgen deprivation therapy (ADT), 73% prior taxane chemotherapy, and 43% prior Lu-177–PSMA RLT. Median baseline PSA was 158–878 ng/mL (Table 1).

**Efficacy Outcomes**:

- **PSA50 Response**: Pooled PSA50 rate was 64% (95% CI: 58–70%), with significant variability across studies (26–91%) (Figure 2).
- **Subgroup Analyses**:
  - Prior therapies: PSA50 rates declined with increasing lines of therapy (78% for 0 vs. 54% for ≥2 lines; *p*<0.0001) (Figure 3).
  - Prior ARPi: Lower PSA50 rates in ARPi-pretreated patients (54% vs. 64.5%; *p*<0.0001) (Figure 4).
  - Visceral metastases: No significant difference in PSA50 rates (68% vs. 71%; *p*=0.23) (Figure 7).
- **Survival**: Median OS ranged from 8–31 months; mPFS ranged from 3–15 months (Table 1).

**Safety Profile**:
Common AEs included anemia (68% any grade), xerostomia (77%), and thrombocytopenia (40%). Severe AEs (grade≥3) were infrequent: anemia (11%), thrombocytopenia (6%), and leukopenia (4%) (Table 3).

**Discussion**
This analysis highlights Ac-225–PSMA TAT as a potent therapeutic option for mCRPC, with PSA50 rates surpassing those of many existing therapies (e.g., ~30–40% for ARPi). The inverse relationship between PSA50 and prior treatment lines suggests greater efficacy in less heavily pretreated patients, possibly due to preserved PSMA expression. Notably, visceral metastases did not diminish response rates, supporting TAT’s applicability in high-risk subgroups.

Safety data indicate manageable toxicity, predominantly hematologic and salivary gland effects. Severe events were rare, contrasting with the higher grade≥3 toxicity rates of chemotherapy (e.g., 20–30% for docetaxel). However, long-term renal and hematologic monitoring remains critical.

**Limitations**: Retrospective design and heterogeneity in dosing (e.g., 100 kBq/kg vs. 8 MBq regimens) limit generalizability. Survival data were inconsistently reported, necessitating cautious interpretation.

**Clinical Implications**: Ac-225–PSMA TAT may be prioritized for patients with limited prior therapies or ARPi resistance. Ongoing phase III trials (e.g., AcTION) will clarify its role in sequencing with Lu-177–PSMA RLT.

**Conclusion**
Ac-225–PSMA TAT achieves robust biochemical responses and tolerable toxicity in advanced mCRPC, particularly in earlier-line settings. Prospective studies should standardize dosing and evaluate combination strategies to optimize outcomes.

**Figures and Tables Referenced**

- Figure 1: CONSORT flowchart (study selection).
- Table 1: Baseline characteristics and outcomes.
- Table 2/Figures 2–7: PSA50 subgroup analyses.
- Table 3: Adverse events.
