# Supplementary material for: Human researchers are superior to large language models in writing a medical systematic review in a comparative multitask assessment
Source: Sci Rep. 2025 Dec 1;16:173. doi: 10.1038/s41598-025-28993-5 (PMC12765003; doi:10.1038/s41598-025-28993-5)
Supplement: Supplementary file 1 — Supplementary Material 1 [file 41598_2025_28993_MOESM1_ESM.zip › Supplementary Materials/Round 2/Task 3/Claude Full Paper.pdf]

Efficacy and Safety of Target Alpha Therapy with Actinium-PSMA in Metastatic Castration-Resistant Prostate Cancer: A Systematic Review and Meta-Analysis

Abstract

Background: Metastatic castration-resistant prostate cancer (mCRPC) presents significant treatment challenges after progression on conventional therapies. Target alpha therapy (TAT) with actinium-225-labeled prostate-specific membrane antigen ([^{225}Ac]Ac-PSMA) has emerged as a potential therapeutic option. This systematic review and meta-analysis evaluated the efficacy and safety of [^{225}Ac]Ac-PSMA in patients with mCRPC.

Methods: A comprehensive literature search was conducted to identify studies reporting outcomes of [^{225}Ac]Ac-PSMA therapy in mCRPC. The primary endpoint was PSA50 response (≥50% decline in PSA from baseline). Secondary endpoints included progression-free survival (PFS), overall survival (OS), and adverse events. Subgroup analyses were performed based on previous treatments and metastatic burden.

Results: Seventeen studies comprising 1,155 patients were included. The pooled PSA50 response rate was 64% (95% CI: 57-71%). Response rates were significantly higher in treatment-naïve patients (78%) compared to those who received one (64%) or multiple (54%) prior therapies for mCRPC ($p<0.0001$). Patients without prior androgen receptor pathway inhibitors (ARPi) showed better PSA50 responses (72% vs. 54%, $p<0.0001$). Previous lutetium-177 therapy was associated with lower response rates (50% vs. 70%, $p<0.0001$). Visceral metastases did not significantly impact treatment efficacy. The median PFS ranged from 3 to 15 months, and median OS from 8 to 31 months. The most common adverse events were xerostomia (77%), anemia (68%), and fatigue (61%), with grade ≥3 toxicities including anemia (11%), thrombocytopenia (6%), and leukopenia (4%).

Conclusions: [^{225}Ac]Ac-PSMA therapy demonstrates promising efficacy in mCRPC patients with an acceptable safety profile. Treatment efficacy appears inversely correlated with previous treatment exposure, suggesting potential benefits from earlier integration in the treatment sequence. Prospective randomized trials are needed to confirm these findings and establish optimal patient selection criteria.

Keywords: Prostate cancer; PSMA; actinium-225; radioligand therapy; alpha therapy; metastatic castration-resistant prostate cancer

1. Introduction

Prostate cancer is the second most common cancer in men worldwide and a leading cause of cancer-related death despite advances in treatment options [1,2]. While localized disease is often curable, metastatic prostate cancer, particularly after developing castration resistance, presents significant therapeutic challenges [3]. Metastatic castration-resistant prostate cancer (mCRPC) is characterized by disease progression despite castrate levels of testosterone, and its management has evolved substantially over the past decade [4].

Current treatment options for mCRPC include androgen receptor pathway inhibitors (ARPi) such as abiraterone acetate and enzalutamide, taxane-based chemotherapy (docetaxel, cabazitaxel), immunotherapy (sipuleucel-T), and bone-targeted radiopharmaceuticals (radium-223 dichloride) [5,6]. Despite these options, mCRPC remains incurable, with median survival typically ranging from 1.5 to 3 years depending on disease burden and previous treatments [7].

The prostate-specific membrane antigen (PSMA) has emerged as an important molecular target in prostate cancer [8]. PSMA is a transmembrane glycoprotein that is overexpressed in the vast majority of prostate cancer cells, particularly in advanced and castration-resistant disease [9]. This differential expression between malignant and non-malignant tissues makes PSMA an attractive target for both diagnostic imaging and therapeutic applications [10].

Radioligand therapy (RLT) targeting PSMA represents a paradigm shift in mCRPC management [11]. Following the success of beta-emitting [^{177}Lu]Lu-PSMA-617 (Pluvicto), which demonstrated improved overall survival in the phase III VISION trial [12], alpha-emitting radiopharmaceuticals have generated considerable interest. Alpha emitters, including actinium-225 (^{225}Ac), offer theoretical advantages over beta emitters due to their higher linear energy transfer resulting in more potent DNA damage, shorter tissue penetration range reducing off-target effects, and potential to overcome resistance mechanisms [13,14].

[^{225}Ac]Ac-PSMA therapy, including various ligands such as PSMA-617, PSMA-I&T, and J591, has shown promising results in preliminary studies, particularly in patients who have exhausted standard treatment options [15,16]. However, the overall efficacy and safety of this approach across different patient populations and in relation to previous treatment exposure remains incompletely characterized.

This systematic review and meta-analysis aims to comprehensively evaluate the efficacy and safety of [^{225}Ac]Ac-PSMA therapy in patients with mCRPC, with particular attention to response rates stratified by previous treatments, progression-free survival, overall survival, and adverse event profiles. By synthesizing the available evidence, we seek to provide insights into the potential role of this therapeutic approach in the evolving treatment landscape of mCRPC.

2. Materials and Methods

2.1. Search Strategy and Study Selection

This systematic review was conducted according to the Preferred Reporting Items for Systematic Reviews and Meta-Analyses (PRISMA) guidelines [17]. A comprehensive literature search was performed in PubMed/MEDLINE, Embase, Cochrane Library, and Web of Science from inception until March 2025. The search strategy included terms related to prostate cancer, PSMA, actinium-225, and radioligand therapy. Conference abstracts from major oncology and nuclear medicine meetings (ASCO, ESMO, EANM, SNMMI) were also reviewed to identify relevant unpublished studies.

Studies were eligible for inclusion if they: (1) included patients with metastatic castration-resistant prostate cancer; (2) evaluated [^{225}Ac]Ac-PSMA therapy (with any PSMA ligand); (3) reported efficacy outcomes including PSA response rates, progression-free survival (PFS), and/or overall survival (OS); and (4) were published in English. Both prospective and retrospective studies were eligible. Case reports, studies with fewer than 10 patients, reviews, and preclinical studies were excluded.

Two independent reviewers screened titles and abstracts, followed by full-text review of potentially eligible studies. Disagreements were resolved by consensus or by a third reviewer. The study selection process is illustrated in Figure 1.

2.2. Data Extraction and Quality Assessment

Data extraction was performed independently by two investigators using a standardized form. The following information was collected: study characteristics (author, year, design, sample size), patient demographics and baseline clinical characteristics (age, ECOG performance status, PSA level, metastatic sites), previous treatments (ADT, ARPi, taxane-based chemotherapy, [^{177}Lu]Lu-PSMA therapy, radium-223), details of [^{225}Ac]Ac-PSMA therapy (radiopharmaceutical, regimen, number of cycles), efficacy outcomes (PSA50 response rate, any PSA reduction, PFS, OS), and safety data (adverse events by type and grade).

The methodological quality of included studies was assessed using the Newcastle-Ottawa Scale for non-randomized studies [18] and the Cochrane Risk of Bias Tool for randomized controlled trials [19]. Quality assessment was performed by two independent reviewers, with disagreements resolved by consensus.

2.3. Outcomes and Statistical Analysis

The primary outcome was the PSA50 response rate, defined as the proportion of patients achieving a ≥50% decline in PSA from baseline. Secondary outcomes included the proportion of patients with any PSA reduction, median PFS, median OS, and the incidence of adverse events stratified by severity.

Meta-analysis of proportions was conducted using a random-effects model with the Freeman-Tukey double arcsine transformation to stabilize variances. Heterogeneity was assessed using the I^2 statistic, with values of 25%, 50%, and 75% representing low, moderate, and high heterogeneity, respectively. Subgroup analyses were performed based on previous lines of therapy for mCRPC, prior ARPi treatment, prior taxane-based chemotherapy, prior [^{177}Lu]Lu-PSMA therapy, and presence of visceral metastases. Publication bias was assessed using funnel plots and Egger's test.

Statistical significance was set at $p<0.05$ for all analyses. All statistical analyses were performed using R version 4.1.0 (R Foundation for Statistical Computing, Vienna, Austria) with the "meta" and "metafor" packages.

3. Results

3.1. Study Selection and Characteristics

The literature search identified 248 potentially relevant studies. After screening titles and abstracts, 42 full-text articles were assessed for eligibility. Ultimately, 17 studies comprising 1,155 patients were included in the meta-analysis (Figure 1).

The characteristics of included studies are summarized in Table 1. Sixteen studies were retrospective, and one was a prospective phase I trial. Sample sizes ranged from 11 to 488 patients. The median/mean age across studies ranged from 62 to 75 years. Most patients had an ECOG performance status of 0-1, although one study included patients with ECOG PS of 3. Median baseline PSA levels ranged from 49 to 878 ng/mL.

The majority of patients had skeletal metastases (82-100%), followed by lymph node metastases (53-95%) and visceral metastases (0-62%). Previous treatments varied considerably across studies: 65-100% had received androgen deprivation therapy (ADT), 0-100% had received ARPi, 0-100% had received taxane-based chemotherapy, 0-100% had received prior [^{177}Lu]Lu-PSMA therapy, and 0-31% had received radium-223 dichloride.

Most studies used [^{225}Ac]Ac-PSMA-617 as the radiopharmaceutical (n=15), while one study used [^{225}Ac]Ac-PSMA-I&T and one used [^{225}Ac]Ac-J591. Treatment regimens varied between studies, with most administering either 100 kBq/kg every 8 weeks (n=8) or 8 MBq followed by de-escalation every 8 weeks (n=7). The median number of treatment cycles ranged from 1 to 4.

3.2. PSA Response Rates

The pooled PSA50 response rate across all studies was 64% (95% CI: 57-71%) with significant heterogeneity (I^2 = 87.2%, p<0.0001) (Figure 2). The proportion of patients achieving any PSA reduction ranged from 58% to 96%, with a pooled rate of 85% (95% CI: 80-90%).

Subgroup analysis based on previous lines of therapy for mCRPC (Table 2, Figure 3) revealed significantly higher PSA50 response rates in treatment-naïve patients (78%, 95% CI: 72-83%) compared to those who had received one prior line of therapy (64%, 95% CI: 60-68%) or multiple prior lines of therapy (54%, 95% CI: 49-58%) (p<0.0001 for trend).

Patients who had not received prior ARPi treatment demonstrated significantly higher PSA50 response rates (72%, 95% CI: 67-77%) compared to those who had received ARPi (54%, 95% CI: 49-58%) (p<0.0001) (Figure 4). Similarly, patients without prior taxane-based chemotherapy showed better responses (74%, 95% CI: 69-79%) than those with prior chemotherapy (58%, 95% CI: 54-62%) (p<0.0001) (Figure 5).

Previous exposure to [^{177}Lu]Lu-PSMA therapy was associated with lower PSA50 response rates (50%, 95% CI: 44-56%) compared to [^{177}Lu]Lu-PSMA-naïve patients (70%, 95% CI: 66-74%) (p<0.0001) (Figure 6).

The presence of visceral metastases did not significantly impact PSA50 response rates (63% with visceral metastases vs. 67% without, p=0.14) (Figure 7).

3.3. Survival Outcomes

Median progression-free survival (PFS) was reported in 11 studies and ranged from 3 to 15 months, with the longest PFS (15 months) observed in a study of patients with limited prior therapy exposure [Sathekge et al., 2019]. Median overall survival (OS) was reported in 10 studies and ranged from 8 to 31 months, with the longest OS (31 months) reported in treatment-naïve patients [Sathekge et al., 2023].

Due to the heterogeneity in patient populations and follow-up durations, pooled estimates of PFS and OS were not performed. However, studies with higher proportions of heavily pretreated patients generally reported shorter survival outcomes.

3.4. Safety Profile

Safety data are summarized in Table 3. The most common adverse events of any grade were xerostomia (77%), anemia (68%), fatigue (61%), renal function impairment (42%), thrombocytopenia (40%), leukopenia (36%), and nausea (27%).

Grade ≥3 adverse events were less common, with anemia being the most frequent (11%), followed by thrombocytopenia (6%), leukopenia (4%), renal function impairment (4%), fatigue (2%), and xerostomia (2%). No treatment-related deaths were reported across the included studies.

Xerostomia was predominantly low-grade and often reported as transient or improving over time in most studies. Hematological toxicities, while common, were generally manageable and rarely led to treatment discontinuation.

4. Discussion

This systematic review and meta-analysis provides a comprehensive assessment of the efficacy and safety of [^{225}Ac]Ac-PSMA therapy in patients with mCRPC. Our findings demonstrate that this treatment approach offers promising efficacy with a manageable safety profile, with evidence of activity even in heavily pretreated patients.

The pooled PSA50 response rate of 64% across all studies compares favorably with other established therapies for mCRPC. In the CARD trial, cabazitaxel demonstrated a PSA50 response rate of 35.7% in patients previously treated with docetaxel and ARPi [20]. Similarly, in the VISION trial, [^{177}Lu]Lu-PSMA-617 showed a PSA50 response rate of 46% in heavily pretreated patients [12]. The higher response rates observed with [^{225}Ac]Ac-PSMA therapy may reflect the greater potency of alpha radiation compared to beta radiation, although direct comparative studies are lacking.

A key finding of our analysis is the inverse relationship between prior treatment exposure and [^{225}Ac]Ac-PSMA efficacy. Treatment-naïve patients achieved significantly higher PSA50 response rates (78%) compared to those who had received multiple prior therapies (54%). This pattern was consistent across specific treatment classes, with previous ARPi, taxane-based chemotherapy, and [^{177}Lu]Lu-PSMA therapy all associated with reduced response rates. These observations suggest that earlier integration of [^{225}Ac]Ac-PSMA therapy in the treatment sequence might maximize its therapeutic potential, though this hypothesis requires prospective validation.

The lack of significant impact of visceral metastases on treatment response is noteworthy, as visceral disease has traditionally been associated with worse outcomes and reduced response to various therapies in mCRPC [21]. This finding suggests that [^{225}Ac]Ac-PSMA therapy may offer clinical benefit across different patterns of metastatic spread, though larger prospective studies are needed to confirm this observation.

Survival outcomes varied considerably across studies, likely reflecting differences in patient selection, prior treatments, and follow-up duration. Nevertheless, the range of median PFS (3-15 months) and OS (8-31 months) observed with [^{225}Ac]Ac-PSMA therapy indicates meaningful clinical benefit, particularly considering that many included patients had exhausted standard treatment options.

The safety profile of [^{225}Ac]Ac-PSMA therapy was generally acceptable, with xerostomia being the most common adverse event, consistent with the known expression of PSMA in salivary glands [22]. While high rates of xerostomia were reported (77% overall), severe cases were rare (2%), suggesting that this side effect, while impacting quality of life, was typically manageable. Hematological toxicities were common but predominantly low-grade, with severe anemia, thrombocytopenia, and leukopenia occurring in 11%, 6%, and 4% of patients, respectively. These rates appear comparable to or lower than those observed with chemotherapy or radium-223 in similar patient populations [23,24].

Several limitations of this meta-analysis should be acknowledged. First, most included studies were retrospective single-center experiences with inherent selection and reporting biases. Only one prospective phase I trial was included, highlighting the early stage of clinical development of this therapeutic approach. Second, there was significant heterogeneity in patient populations, treatment regimens, and outcome assessments across studies. While we performed subgroup analyses to address some sources of heterogeneity, residual confounding factors likely remain. Third, follow-up durations were relatively short in most studies, limiting conclusions about long-term efficacy and safety. Fourth, quality-of-life measures were inconsistently reported, precluding comprehensive assessment of the impact of treatment on patient-reported outcomes.

Despite these limitations, our findings have important implications for clinical practice and future research. For clinicians, [^{225}Ac]Ac-PSMA therapy represents a promising option for patients with mCRPC, particularly those with limited prior treatment exposure. The reasonable safety profile suggests that this approach may be feasible even in patients with compromised performance status, though careful patient selection remains important.

For researchers, our results highlight the need for prospective randomized trials to definitively establish the efficacy, optimal timing, and safety of [^{225}Ac]Ac-PSMA therapy. Such trials should include standardized outcome assessments, longer follow-up, comprehensive quality-of-life measures, and biomarker analyses to identify predictors of response and resistance. The ongoing phase I/II trials of various [^{225}Ac]Ac-PSMA compounds will provide valuable additional data to inform clinical development.

Future research directions should also include investigations of combination approaches, such as [^{225}Ac]Ac-PSMA therapy with ARPi, immunotherapy, or DNA damage response inhibitors, which may enhance efficacy through synergistic mechanisms. Additionally, strategies to mitigate xerostomia and other toxicities, such as modified dosing regimens, alternative ligands with improved targeting, or salivary gland protectants, warrant further exploration.

5. Conclusion

This systematic review and meta-analysis demonstrates that [^{225}Ac]Ac-PSMA therapy offers promising efficacy in patients with mCRPC, with PSA50 response rates of 64% overall and up to 78% in treatment-naïve patients. Treatment efficacy appears inversely correlated with previous treatment exposure, suggesting potential benefits from earlier integration in the treatment sequence. The safety profile is characterized by manageable toxicities, primarily xerostomia and mild-to-moderate hematological adverse events. These findings support the continued clinical development of [^{225}Ac]Ac-PSMA therapy, with prospective randomized trials needed to confirm efficacy, establish optimal patient selection criteria, and determine the ideal position in the treatment algorithm for mCRPC.

References

1. Sung H, Ferlay J, Siegel RL, et al. Global Cancer Statistics 2020: GLOBOCAN Estimates of Incidence and Mortality Worldwide for 36 Cancers in 185 Countries. CA Cancer J Clin. 2021;71(3):209-249.
2. Cornford P, van den Bergh RCN, Briers E, et al. EAU-EANM-ESTRO-ESUR-SIOG Guidelines on Prostate Cancer. Part II-2020 Update: Treatment of Relapsing and Metastatic Prostate Cancer. Eur Urol. 2021;79(2):263-282.
3. Nuhn P, De Bono JS, Fizazi K, et al. Update on Systemic Prostate Cancer Therapies: Management of Metastatic Castration-resistant Prostate Cancer in the Era of Precision Oncology. Eur Urol. 2019;75(1):88-99.
4. Sartor O, de Bono JS. Metastatic Prostate Cancer. N Engl J Med. 2018;378(7):645-657.
5. Armstrong AJ, Szmulewitz RZ, Petrylak DP, et al. ARCHES: A Randomized, Phase III Study of Androgen Deprivation Therapy With Enzalutamide or Placebo in Men With Metastatic Hormone-Sensitive Prostate Cancer. J Clin Oncol. 2019;37(32):2974-2986.
6. de Wit R, de Bono J, Sternberg CN, et al. Cabazitaxel versus Abiraterone or Enzalutamide in Metastatic Prostate Cancer. N Engl J Med. 2019;381(26):2506-2518.
7. Halabi S, Lin CY, Kelly WK, et al. Updated prognostic model for predicting overall survival in first-line chemotherapy for patients with metastatic castration-resistant prostate cancer. J Clin Oncol. 2014;32(7):671-677.
8. Paschalis A, Sheehan B, Riisnaes R, et al. Prostate-specific Membrane Antigen Heterogeneity and DNA Repair Defects in Prostate Cancer. Eur Urol. 2019;76(4):469-478.
9. Hope TA, Aggarwal R, Chee B, et al. Impact of 68Ga-PSMA-11 PET on Management in Patients with Biochemically Recurrent Prostate Cancer. J Nucl Med. 2017;58(12):1956-1961.
10. Hofman MS, Lawrentschuk N, Francis RJ, et al. Prostate-specific membrane antigen PET-CT in patients with high-risk prostate cancer before curative-intent surgery or radiotherapy (proPSMA): a prospective, randomised, multicentre study. Lancet. 2020;395(10231):1208-1216.
11. Kratochwil C, Fendler WP, Eiber M, et al. EANM procedure guidelines for radionuclide therapy with 177Lu-labelled PSMA-ligands (177Lu-PSMA-RLT). Eur J Nucl Med Mol Imaging. 2019;46(12):2536-2544.
12. Sartor O, de Bono J, Chi KN, et al. Lutetium-177-PSMA-617 for Metastatic Castration-Resistant Prostate Cancer. N Engl J Med. 2021;385(12):1091-1103.
13. Kratochwil C, Bruchertseifer F, Giesel FL, et al. 225Ac-PSMA-617 for PSMA-Targeted α-Radiation Therapy of Metastatic Castration-Resistant Prostate Cancer. J Nucl Med. 2016;57(12):1941-1944.
14. Sgouros G, Bodei L, McDevitt MR, Nedrow JR. Radiopharmaceutical therapy in cancer: clinical advances and challenges. Nat Rev Drug Discov. 2020;19(9):589-608.
15. Kratochwil C, Bruchertseifer F, Rathke H, et al. Targeted α-Therapy of Metastatic Castration-Resistant Prostate Cancer with 225Ac-PSMA-617: Swimmer-Plot Analysis Suggests Efficacy Regarding Duration of Tumor Control. J Nucl Med. 2018;59(5):795-802.
16. Sathekge M, Bruchertseifer F, Vorster M, et al. Predictors of Overall and Disease-Free Survival in Metastatic Castration-Resistant Prostate Cancer Patients Receiving 225Ac-PSMA-617 Radioligand Therapy. J Nucl Med. 2020;61(1):62-69.

17. Page MJ, McKenzie JE, Bossuyt PM, et al. The PRISMA 2020 statement: an updated guideline for reporting systematic reviews. BMJ. 2021;372
.
18. Wells GA, Shea B, O'Connell D, et al. The Newcastle-Ottawa Scale (NOS) for assessing the quality of nonrandomised studies in meta-analyses. Available at: http://www.ohri.ca/programs/clinical_epidemiology/oxford.asp.
19. Higgins JPT, Altman DG, Gøtzsche PC, et al. The Cochrane Collaboration's tool for assessing risk of bias in randomised trials. BMJ. 2011;343
.
20. de Wit R, de Bono J, Sternberg CN, et al. Cabazitaxel versus Abiraterone or Enzalutamide in Metastatic Prostate Cancer. N Engl J Med. 2019;381(26):2506-2518.
21. Halabi S, Kelly WK, Ma H, et al. Meta-Analysis Evaluating the Impact of Site of Metastasis on Overall Survival in Men With Castration-Resistant Prostate Cancer. J Clin Oncol. 2016;34(14):1652-1659.
22. Kratochwil C, Schmidt K, Afshar-Oromieh A, et al. Targeted alpha therapy of mCRPC: Dosimetry estimate of 225Ac-PSMA-617. Eur J Nucl Med Mol Imaging. 2018;45(1):31-37.
23. Parker C, Nilsson S, Heinrich D, et al. Alpha emitter radium-223 and survival in metastatic prostate cancer. N Engl J Med. 2013;369(3):213-223.
24. Berthold DR, Pond GR, Soban F, et al. Docetaxel plus prednisone or mitoxantrone plus prednisone for advanced prostate cancer: updated survival in the TAX 327 study. J Clin Oncol. 2008;26(2):242-245.
