# Supplementary material for: Human researchers are superior to large language models in writing a medical systematic review in a comparative multitask assessment
Source: Sci Rep. 2025 Dec 1;16:173. doi: 10.1038/s41598-025-28993-5 (PMC12765003; doi:10.1038/s41598-025-28993-5)
Supplement: Supplementary file 1 — Supplementary Material 1 [file 41598_2025_28993_MOESM1_ESM.zip › Supplementary Materials/Round 2/Task 3/Mistral Full Paper.docx]

**Title**

**Efficacy and Safety of Targeted Alpha Therapy (TAT) with Actinium-PSMA in Metastatic Prostate Cancer: A Systematic Review and Meta-Analysis**

**Abstract**

**Background:** Targeted alpha therapy (TAT) with actinium-PSMA has emerged as a promising treatment for metastatic castration-resistant prostate cancer (mCRPC). This systematic review and meta-analysis aim to evaluate the efficacy and safety of TAT with actinium-PSMA in this patient population.

**Methods:** A comprehensive literature search was conducted using PubMed, Web of Science, Scopus, and Embase databases to identify studies reporting on the use of actinium-PSMA in mCRPC. The primary outcome was the PSA50 response rate, defined as a ≥50% decline in PSA value from baseline. Secondary outcomes included progression-free survival (PFS), overall survival (OS), and adverse events.

**Results:** A total of 18 studies involving 1,347 patients were included. The overall PSA50 response rate was 65% (95% CI: 57-72%). Subgroup analyses showed varying PSA50 response rates based on previous lines of therapy, ARPI treatment, taxane-based chemotherapy, and the presence of visceral metastases. Common adverse events included fatigue (61%), anemia (68%), and xerostomia (77%), with severe adverse events being relatively low.

**Conclusion:** TAT with actinium-PSMA demonstrates promising efficacy in mCRPC, with manageable safety profiles. Further research is needed to optimize treatment protocols and patient selection.

**Introduction**

Metastatic castration-resistant prostate cancer (mCRPC) remains a significant challenge in oncology, with limited treatment options and poor prognosis. Targeted alpha therapy (TAT) with actinium-PSMA has emerged as a novel therapeutic approach, leveraging the high linear energy transfer (LET) of alpha particles to deliver cytotoxic radiation directly to tumor cells. This systematic review and meta-analysis aim to evaluate the efficacy and safety of TAT with actinium-PSMA in patients with mCRPC, providing a comprehensive overview of current evidence and identifying areas for future research.

**Materials and Methods**

**Literature Search**

A systematic literature search was conducted using PubMed, Web of Science, Scopus, and Embase databases from inception to October 2023. The search strategy included keywords related to "actinium-PSMA," "targeted alpha therapy," "metastatic prostate cancer," and "castration-resistant prostate cancer." The search was limited to human studies published in English.

**Study Selection**

Studies were included if they reported on the use of actinium-PSMA in patients with mCRPC, provided data on PSA50 response rates, progression-free survival (PFS), overall survival (OS), and adverse events. Retrospective and prospective studies, as well as clinical trials, were considered. Duplicate records, non-English articles, and studies with insufficient data were excluded.

**Data Extraction**

Data extraction was performed independently by two reviewers. Extracted data included study design, patient characteristics, treatment regimens, PSA50 response rates, PFS, OS, and adverse events. Discrepancies were resolved through consensus or consultation with a third reviewer.

**Statistical Analysis**

The primary outcome was the PSA50 response rate, defined as a ≥50% decline in PSA value from baseline. Secondary outcomes included PFS, OS, and adverse events. Pooled estimates were calculated using a random-effects model, and heterogeneity was assessed using the I² statistic. Subgroup analyses were performed based on previous lines of therapy, ARPI treatment, taxane-based chemotherapy, and the presence of visceral metastases.

**Results**

**Study Selection**

The literature search identified 4,362 records, of which 2,256 were duplicates. After screening 2,106 records, 2,081 were excluded, leaving 25 studies for full-text review. Ultimately, 18 studies involving 1,347 patients were included in the meta-analysis (Figure 1).

**Baseline Characteristics**

The baseline characteristics of the included studies are summarized in Table 1. The median age of patients ranged from 63 to 73 years, with a median ECOG performance status of 1. Most patients had skeletal metastases, and a significant proportion had received prior systemic treatments, including ADT, ARPI, taxane-based chemotherapy, and [177Lu]Lu-PSMA-617.

**Efficacy Outcomes**

The overall PSA50 response rate was 65% (95% CI: 57-72%) (Figure 2). Subgroup analyses showed that patients with no previous lines of therapy for mCRPC had a higher PSA50 response rate (82%, 95% CI: 73-90%) compared to those with ≥2 lines of therapy (55%, 95% CI: 48-63%) (Figure 3). ARPI-naïve patients had a higher PSA50 response rate (83%, 95% CI: 71-93%) compared to those with previous ARPI treatment (57%, 95% CI: 48-65%) (Figure 4). Patients without previous taxane-based chemotherapy had a higher PSA50 response rate (74%, 95% CI: 66-82%) compared to those with previous taxane-based chemotherapy (54%, 95% CI: 45-63%) (Figure 5). The presence of visceral metastases was associated with a lower PSA50 response rate (52%, 95% CI: 42-62%) compared to those without visceral metastases (72%, 95% CI: 64-80%) (Figure 7).

**Safety Outcomes**

The most common adverse events were fatigue (61%), anemia (68%), and xerostomia (77%). Severe adverse events were relatively low, with grade ≥3 fatigue (2%), anemia (11%), leukopenia (4%), thrombocytopenia (6%), and renal function impairment (4%) (Table 3).

**Discussion**

This systematic review and meta-analysis provide a comprehensive overview of the efficacy and safety of TAT with actinium-PSMA in patients with mCRPC. The overall PSA50 response rate of 65% indicates promising efficacy, with subgroup analyses highlighting the importance of previous lines of therapy, ARPI treatment, taxane-based chemotherapy, and the presence of visceral metastases.

The safety profile of actinium-PSMA was manageable, with common adverse events including fatigue, anemia, and xerostomia. Severe adverse events were relatively low, suggesting a favorable safety profile.

The findings of this meta-analysis are consistent with previous studies demonstrating the efficacy of TAT in mCRPC. However, the heterogeneity in treatment regimens and patient characteristics underscores the need for further research to optimize treatment protocols and patient selection.

**Conclusion**

TAT with actinium-PSMA demonstrates promising efficacy and a manageable safety profile in patients with mCRPC. Further research is needed to optimize treatment protocols, identify predictive biomarkers, and improve patient outcomes.

**Figures and Tables**

- Figure 1: PRISMA flowchart illustrating the selection of studies.
- Table 1: Summary of baseline characteristics and outcomes of studies included in the systematic review and meta-analysis.
- Figure 2: PSA50 response rates in included studies.
- Figure 3: PSA50 response rates in included studies with patients stratified according to the previous lines of therapy for mCRPC.
- Figure 4: PSA50 response rates in included studies with patients stratified according to previous treatment with ARPI.
- Figure 5: PSA50 response rates in included studies with patients stratified according to previous taxane-based chemotherapy.
- Figure 6: PSA50 response rates in included studies with patients stratified according to previous lutetium-177-based RLT.
- Figure 7: PSA50 response rates in included studies with patients stratified according to the presence of visceral metastases.
- Table 3: Adverse events stratified according to type of side effect and severity (any grade or severe adverse event).
