# Supplementary material for: Human researchers are superior to large language models in writing a medical systematic review in a comparative multitask assessment
Source: Sci Rep. 2025 Dec 1;16:173. doi: 10.1038/s41598-025-28993-5 (PMC12765003; doi:10.1038/s41598-025-28993-5)
Supplement: Supplementary file 1 — Supplementary Material 1 [file 41598_2025_28993_MOESM1_ESM.zip › Supplementary Materials/Round 2/Task 1/Database Search Updated.docx]

**Risultati 21/04/2025**

**ChatGPT (o4-mini-high)**

9 correct papers; no more hallucinated papers or papers not meeting the inclusion or exclusion criteria.

**Gemini (2.5 Deep Research)**

13 papers meeting inclusion and exclusion criteria; the generated table contained some mistakes on titles, publication year, or author’s name but DOIs were correct.

**Claude (3.7 Sonnet with Extended Thinking)**

Explicitely states it can not complete the task as it does not have access to PubMed or other similar web databases.

**DeepSeek (R1+search)**

2 correct papers, search stopped midway through because “server is busy”

**Mistral Le Chat**

3 correct papers

2 papers not meeting inclusion criteria (1 preclinical, 1 on lutetium-PSMA)

**Grok 3 beta**

8 correct papers

1 paper missing or hallucinating details

**Precedenti risultati**

**ChatGPT (o3-mini-high)**

8 correct papers

1 papers not meeting the inclusion criteria

10 hallucinated papers

(note: several attempts at obtaining a list of more than 3-4 papers; these short lists did include however all correct papers).

**Gemini (2.0 Flash Thinking Experimental with Search)**

30 hallucinated papers

(note: several attempts at obtaining a list of more than 3-4 papers; these short list did also include reviews or papers not meeting inclusion criteria)

**Claude (3.7 Sonnet with Extended Thinking)**

Explicitely states it can not complete the task as it does not have access to PubMed or other similar web databases.

**DeepSeek (R1+search)**

5 correct papers

25 papers not meeting inclusion criteria (9 reviews including our preprint on Theranostics, 1 study design, 1 preclinical paper, 5 off-topic paper, 1 divulgative article, 1 editorial, 7 duplicated correct results)

3 hallucinated papers

**Mistral Le Chat**

2 correct papers

7 papers not meeting inclusion criteria (4 reviews, 2 preclinical papers, 1 editorial)
