# Supplementary material for: Human researchers are superior to large language models in writing a medical systematic review in a comparative multitask assessment
Source: Sci Rep. 2025 Dec 1;16:173. doi: 10.1038/s41598-025-28993-5 (PMC12765003; doi:10.1038/s41598-025-28993-5)
Supplement: Supplementary file 2 — Supplementary Material 2 [file 41598_2025_28993_MOESM2_ESM.docx]

| **Round 1** |  | **Title** | **Abstract** | | **Introduction** | **Methods** | **Results** | **Discussion** | **References** | **Notes** |
| --- | --- | --- | --- | --- | --- | --- | --- | --- | --- | --- |
| **ChatGPT** | **Evaluation** | **A** | **P.A.** | | **P.A.** | **I** | **I** | **I** | **I** | Tables and figures were provided; a note was included for sections related to editorial requirements |
|  | **Comment** |  | Structured; only mentions mCRPC, incorrect number of included studies | | Short, only contextualises for mCRPC, clear aim of the review but no mention of meta-analysis | Vague and imprecise, misses key details about selection process, quality assessment and statistics | Vague and imprecise, incorrect number of included studies, misses key details and the meta-analysis | Only contextualises for mCRPC, misses key discussion details, no comparison with literature data, mention of missing meta-analysis results | Presence of a note |  |
|  | **Length** | 18 total word count | 311 total word count | | 194 total word count | 256 total word count | 422 total word count | 458 total word count | 0 |  |
| **Claude** | **Evaluation** | **P.A.** | **P.A.** | | **P.A.** | **P.A.** | **P.A.** | **P.A.** | **P.A.** | Tables and figures were provided |
|  | **Comment** | Only mentions mCRPC | Only mentions mCRPC; incorrect number of included studies | | Only contextualises for mCRPC | Only mentions mCRPC, misses some details about statistics | Only mentions mCRPC; incorrect number of included studies; fails to detail meta-analysis | Only contextualises for mCRPC | A few hallucinated references |  |
|  | **Length** | 19 total word count | 267 total word count | | 455 total word count | 565 total word count | 583 total word count | 912 total word count | 21 records |  |
| **Gemini** | **Evaluation** | **P.A.** | **P.A.** | | **P.A.** | **I** | **P.A.** | **P.A.** | **I** | Tables and figures were provided together with captions |
|  | **Comment** | Only mentions mCRPC | Only mentions mCRPC | | Only contextualises for mCRPC | Vague and imprecise, misses details about quality assessment and statistics | Only mentions mCRPC | Only contextualises for mCRPC | Not provided |  |
|  | **Length** | 15 total word count | 458 total word count | | 247 total word count | 344 total word count | 485 total word count | 544 total word count | 0 |  |
| **DeepSeek** | **Evaluation** | **P.A.** | **P.A.** | | **I** | **I** | **I** | **I** | **I** | Tables and figures were referenced together with captions. Conflict of interest and funding statements were provided |
|  | **Comment** | Only mentions mCRPC | Only mentions mCRPC; incorrect number of included studies | | Short and uninformative, only contextualises for mCRPC | Vague and imprecise, misses several key details about selection process, outcomes, and statistics | Only mentions mCRPC; incorrect number of studies included; severely lacking key details | Only contextualises for mCRPC, severely lacking key discussion points, no comparison with literature data | Not provided |  |
|  | **Length** | 19 total word count | 185 total word count | | 81 total word count | 123 total word count | 123 total word count | 160 total word count | 0 |  |
| **Mistral** | **Evaluation** | **A** | **P.A.** | | **I** | **I** | **I** | **I** | **I** | None |
|  | **Comment** |  | Not mention meta-analysis methods | | Short and uninformative, only contextualises for mCRPC | Misses key details especially for statistics | Vague and lacking key details, does not mention meta-analysis | Severely lacking key discussion points, no comparison with literature data | Not provided |  |
|  | **Length** | 18 total word count | 258 total word count | 258 total word count | | 256 total word count | 233 total word count | 177 total word count | 0 |  |

| **Round 2** |  | **Title** | **Abstract** | **Introduction** | **Methods** | **Results** | **Discussion** | **References** | **Notes** |
| --- | --- | --- | --- | --- | --- | --- | --- | --- | --- |
| **ChatGPT** | **Evaluation** | **I** | **P.A.** | **I** | **I** | **I** | **I** | **I** | None |
|  | **Comment** | Not provided | Only mentions mCRPC; incorrect number of included studies | Short and uninformative, only contextualises for mCRPC | Vague and imprecise, severely lacking key details | Severely lacking key details; incorrect number of included studies | Only contextualises for mCRPC, misses several key discussion details | Not provided, presence of a note |  |
|  | **Length** | 0 total word count | 224 total word count | 62 total word count | 74 total word count | 123 total word count | 154 total word count | 0 |  |
| **Claude** | **Evaluation** | **P.A.** | **P.A.** | **P.A.** | **P.A.** | **P.A.** | **P.A.** | **P.A.** | No tables and figures were provided; non-hallucinated references were provided |
|  | **Comment** | Only mentions mCRPC | Only mentions mCRPC; incorrect number of included studies | Only contextualises for mCRPC | Vague methodology description for statistics | Incorrect number of included studies; fails to detail meta-analysis | Only contextualises for mCRPC | A few hallucinated references |  |
|  | **Length** | 19 total word count | 283 total word count | 373 total word count | 459 total word count | 628 total word count | 790 total word count | 24 records |  |
| **Gemini** | **Evaluation** | **P.A.** | **P.A.** | **P.A.** | **I** | **P.A.** | **P.A.** | **I** | Gemini provided a note specifying the “methods” section needs to be integrated with specific details by the user; a note was included for sections related to editorial requirements and for references |
|  | **Comment** | Only mentions mCRPC | Only mentions mCRPC | Short, only contextualises for mCRPC | Vague and imprecise, misses key details | Only mentions mCRPC | Only contextualises for mCRPC | Not provided |  |
|  | **Length** | 19 total word count | 345 total word count | 215 total word count | 215 total word count | 456 total word count | 440 total word count | 0 |  |
| **DeepSeek** | **Evaluation** | **P.A.** | **P.A.** | **I** | **I** | **I** | **I** | **I** | Tables and figures were referenced together with captions |
|  | **Comment** | Only mentions mCRPC | Only mentions mCRPC; incorrect number of included studies | Short and uninformative | Vague and imprecise, misses several key details about selection process, outcomes and statistics | Severely lacking key details | Only contextualises for mCRPC, severely lacking key discussion points, no comparison with literature data | Not provided |  |
|  | **Length** | 19 total word count | 183 total word count | 88 total word count | 118 total word count | 158 total word count | 188 total word count | 0 |  |
| **Mistral** | **Evaluation** | **A** | **P.A.** | **I** | **I** | **I** | **I** | **I** | Tables and figures were referenced together with captions |
|  | **Comment** |  | Inappropriate background, does not mention meta-analysis | Inappropriately short and uninformative, only contextualises for mCRPC | Does not mention meta-analysis, misses key details | Vague and lacking key details, does not mention meta-analysis | Severely lacking key discussion points | Not provided |  |
|  | **Length** | 19 total word count | 182 total word count | 82 total word count | 206 total word count | 265 total word count | 149 total word count | 0 |  |
| **Grok** | **Evaluation** | **A** | **P.A.** | **P.A.** | **I** | **I** | **I** | **I** | None |
|  | **Comment** |  | Only mentions mCRPC | Only contextualises for mCRPC | Misses key details about statistics | Vague and lacking key details, does not mention meta-analysis | Severely lacking key discussion points, no comparison with literature data | Not provided |  |
|  | **Length** | 18 total word count | 250 total word count | 197 total word count | 263 total word count | 332 total word count | 318 total word count | 0 |  |

**Supplementary Table 1**. Task 3 evaluation results, with comments and length details. A: appropriate; P.A.: partially appropriate; I: inappropriate. mCRPC: metastatic castration-resistant prostate cancer.
