# Supplementary material for: Human researchers are superior to large language models in writing a medical systematic review in a comparative multitask assessment
Source: Sci Rep. 2025 Dec 1;16:173. doi: 10.1038/s41598-025-28993-5 (PMC12765003; doi:10.1038/s41598-025-28993-5)
Supplement: Supplementary file 3 — Supplementary Material 3 [file 41598_2025_28993_MOESM3_ESM.docx]

Following are the initial identical prompts provided to each LLM for each Task.

**Task 1**

I am performing a systematic review of the literature. My review focuses on radioligand therapy with actinium-psma in prostate cancer. I want you to search PubMed for articles on actinium-psma, scan the abstracts, then apply the following exclusion criteria to exclude articles: a) review articles, meta-analyses, guidelines, case reports, case series, editorials, book chapters, and conference abstracts; (b) studies with outcomes available for fewer than ten patients; (c) preclinical studies not involving human subjects; (d) articles not in the English language; (e) studies on PSMA-targeted alpha/beta combined/tandem therapies. Then, list me the resulting articles with their Title, Publication Year, First Author, Journal, and DOI.

**Task 2**

I am performing a systematic review of the literature on therapy with actinium-psma. I selected 18 papers to be included in the review. I will send you the full papers in the following messages. I want you to analyze them and generate a table, listing for each article: - Study design (retrospective or prospective) - Number of patients treated with actinium-psma only (not counting patients undergoing cocktail treatment with both actiunium-psma and lutetium-psma) - Mean or median age of included patients, if the datum is available - Mean or median ECOG PS, if the datum is available - Median baseline PSA - Percentages of included patients with bone, lymph node, or visceral metastases, if data are available - Percentages of included patients previously treated with ADT, ARPI/ARSI, taxane-based chemotherapy, lutetium-psma, or radium-223, if data are availabe - exact type of actinium-psma radiopharmaceutical employed and dose/treatment regimen - Median number of treatment cycles - Median follow-up time, if the datum is available - Main results in terms of PSA50, Any PSA reduction, median PFS, and median OS, if data are available

**Task 3**

I am writing a systematic review and meta-analysis of the literature on the efficacy and safety of target alpha therapy (TAT) with actinium-PSMA in metastatic prostate cancer. I already performed the literature search, article selection, and data analyses. In attachment you will find the tables and figures of the main results of the article selection process and data analyses. I want you to produce the full text of the scientific paper for this systematic review and meta-analysis. Please adhere to the usual format for scientific paper in general and systematic reviews in particular. Accordingly, the full paper should include the Title, an Abstract, the Introduction section detailing the current scientific background and the aim of our paper, the Materials and Methods section, the Results section, and the Discussion commenting on our results and contextualizing them.

Below, the initial prompt provided to the custom GPT available at <https://chatgpt.com/g/g-aVMV7ejsa-systematic-review-assistant> for the ROBINS-E subtask analysis of Task 2.

**Subtask**

I am performing a systematic review of the literature on therapy with actinium-psma. I selected 18 papers to be included in the review. I will send you the full papers in the following messages. I want you to analyze them and perform for each of them the complete ROBINS-E analysis.
